# Sodium-glucose cotransporter 2 inhibitor-associated perioperative ketoacidosis: a systematic review of case reports

**DOI:** 10.1007/s00540-023-03174-8

**Published:** 2023-02-27

**Authors:** Hiroyuki Seki, Satoshi Ideno, Toshiya Shiga, Hidenobu Watanabe, Motoaki Ono, Akira Motoyasu, Hikari Noguchi, Kazuya Kondo, Takahiro Yoshikawa, Hiroshi Hoshijima, Shunsuke Hyuga, Miho Shishii, Ai Nagai, Midoriko Higashi, Takashi Ouchi, Kazuki Yasuda, Norifumi Kuratani

**Affiliations:** 1 Department of Anesthesiology, Kyorin University School of Medicine, 6-20-2 Shinkawa Mitaka, Tokyo, 181-8611 Japan; 2 Department of Anesthesiology, Kawasaki Municipal Hospital, Kanagawa, Japan; 3 Department of Anesthesiology, School of Medicine, International University of Health and Welfare, Chiba, Japan; 4 Division of Dento-Oral Anesthesiology, Tohoku University Graduate School of Dentistry, Miyagi, Japan; 5 Department of Anesthesiology, Kitasato University School of Medicine, Kanagawa, Japan; 6 Department of Anesthesiology and Critical Care Medicine, Graduate School of Medical Sciences, Kyushu University, Fukuoka, Japan; 7 Department of Anesthesiology, Tokyo Dental College Ichikawa General Hospital, Chiba, Japan; 8 Department of Diabetes, Endocrinology and Metabolism, Kyorin University School of Medicine, Tokyo, Japan; 9 Department of Anesthesia, Saitama Children’s Medical Center, Saitama, Japan

**Keywords:** Sodium-glucose cotransporter 2 inhibitors, Ketoacidosis, Diabetic ketoacidosis, Systematic review, Case reports

## Abstract

Although the recommended preoperative cessation period for sodium-glucose cotransporter 2 inhibitors (SGLT2is) changed in 2020 (from 24 h to 3–4 days preoperatively) to reduce the risk of SGLT2i-associated perioperative ketoacidosis (SAPKA), the validity of the new recommendation has not been verified. Using case reports, we assessed the new recommendation effectiveness and extrapolated precipitating factors for SAPKA. We searched electronic databases up to June 1, 2022 to assess SAPKA (blood pH < 7.3 and blood or urine ketone positivity within 30 days postoperatively in patients taking SGLT2i). We included 76 publications with 99 cases. The preoperative SGLT2i cessation duration was reported for 59 patients (59.6%). In all cases with available cessation periods, the SGLT2is were interrupted < 3 days preoperatively. No SAPKA cases with > 2-day preoperative cessation periods were found. Many case reports lack important information for estimating precipitating factors, including preoperative SGLT2i cessation period, body mass index, baseline hemoglobin A1c level, details of perioperative fluid management, and type of anesthesia. Our study suggested that preoperative SGLT2i cessation for at least 3 days could prevent SAPKA. Large prospective epidemiologic studies are needed to identify risk factors for SAPKA.

**Supplementary Information:**

The online version contains supplementary material available at 10.1007/s00540-023-03174-8.

## Introduction

Sodium-glucose cotransporter 2 inhibitors (SGLT2is) are a relatively new class of anti-diabetic drugs. They lower blood glucose levels by promoting urinary glucose excretion via the inhibition of SGLT2, expressed in the proximal tubules. Their beneficial effects include reduced hemoglobin A1c (HbA1c) levels, weight loss, and a reduction in blood pressure [1]. Furthermore, recent large clinical trials have demonstrated that they have cardio- and reno-protective effects [2], prompting the US Food and Drug Administration (FDA) to approve them for treating heart failure and chronic kidney diseases, even in patients without diabetes [3, 4]. Available data demonstrate a good tolerability profile for SGLT2i with minor side effects such as genital mycotic infections, urinary tract infections, and increased urination [5]. However, they cause ketoacidosis, a rare but potentially life-threatening side effect. Over the last few years, many patients treated with SGLT2i have reportedly developed severe ketoacidosis [6]. Previous studies have suggested that surgery is a precipitating factor for developing SGLT2i-associated perioperative ketoacidosis (SAPKA), which is attributed to perioperative fasting and metabolic changes caused by surgical stress [7, 8]. In March 2020, the US FDA approved a label change to promote SGLT2i interruption before elective surgery, recommending a longer period (at least 3 days for canagliflozin, dapagliflozin, and empagliflozin, and 4 days for ertugliflozin) than that recommended previously (24 h preoperatively) to reduce the risk of ketoacidosis [9]. However, it is unclear whether this label change is effective in preventing SAPKA because no studies have validated the new recommendation. Furthermore, risk factors for SAPKA have not been completely elucidated given its low incidence. The primary objective of this study was to evaluate the validity of the updated recommendation concerning preoperative cessation of SGLT2is by summarizing the preoperative withholding period in a series of case reports. The secondary objective was to identify risk factors for SAPKA by summarizing the available data. As no clinical trials aiming to determine the potential adverse effects of this class of drug have been published, we conducted this study as a systematic review of case reports.

## Methods

This study was conducted according to the Preferred Reporting Items for Systematic Reviews and Meta-Analyses (PRISMA) 2020 statement [10]. The protocol was published as a preprint before study initiation (https://www.medrxiv.org/content/10.1101/2022.05.22.22275348v1).

### Eligibility criteria

We included case reports or series describing patients receiving SGLT2i who developed ketoacidosis (defined as blood pH < 7.3 and blood or urine ketone positivity) pre-, intra-, or postoperatively up to 30 days. Letters, reviews, and conference abstracts were also included. Patients who were newly prescribed SGLT2i postoperatively and those who did not fulfill the ketoacidosis criteria were excluded. Cases in which the pH value was unclear, but the authors stated “acidosis” were included.

### Search strategy and study selection

Two independent researchers (H.S. and S.I.) searched electronic databases, including PubMed, EMBASE, and Web of Science, up to June 1, 2022, with no language restrictions for identifying perioperative ketoacidosis associated with SGLT2is. The full search strategy is summarized in Supplementary Table S1. The reference lists of all identified studies and those of previous meta-analyses on similar topics were checked. Two reviewers (H.S. and S.I.) independently screened the obtained references’ titles and abstracts and collected full-text articles if potentially relevant. Disagreements were resolved through discussion or consultation with a third author (K.Y.).

### Data extraction

Fourteen reviewers (H.S., S.I., H.W., M.O., A.M., H.N., K.K., T.Y., H.H., S.H., M.S., A.I., M.H., and T.O.) working in seven teams independently extracted data on age, sex, body mass index (BMI), the purpose of SGLT2i prescription, comorbidities, diabetes type, SGLT2i type, anti-diabetic and other medications, surgery and anesthesia type, data on withholding agents preoperatively, nature of presentation as ketoacidosis, biochemical parameters (including serum glucose, HbA1c, pH, serum bicarbonate [HCO_3_], partial pressure of arterial carbon dioxide [PaCO_2_], anion gap [AG], plasma and urine ketones, and glycosuria), management details, complications, and outcomes.

### Assessment of methodological quality of the cases

The methodological quality of the cases was assessed using a previously published method (Supplementary Table S2) [11]. Each case was assessed in five domains, for which a binary (Yes/No) response was generated. The quality of the report was graded from 0 (low) to 5 (high). Disagreements were resolved through discussion or consultation with a third author (K.Y.).

### Statistical analysis

Since this was a systematic review of case reports, descriptive epidemiology was performed on predetermined items for each case. Standard descriptive statistics were performed on those reported numerically, and pooled analysis was not performed. For continuous variables with a normal distribution, the mean (standard deviation) was reported. For non-normally distributed variables, the median (range) was reported. Data were analyzed using the PRISM 6 software (GraphPad Software, Inc., CA).

## Results

**Fig. 1:**
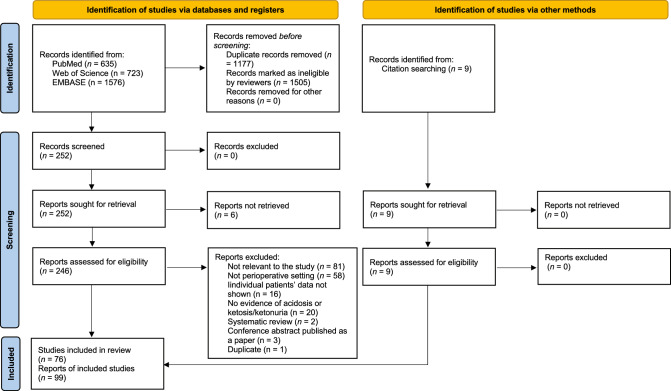
PRISMA 2020 flow diagram

**Fig. 2:**
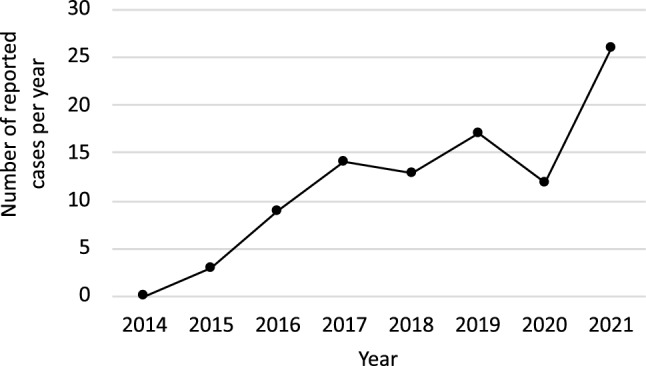
Number of reported cases of SGLT2i-associated perioperative ketoacidosis. SGLT2i, sodium-glucose cotransporter 2 inhibitor

This study was conducted according to the pre-published protocol. The search strategy identified 2934 citations, among which 252 were assessed in full-text; 76 publications with 99 cases were included in the review (Fig. 1) [12–87]. Eighty-one cases included in this study were reported in published papers, and the remaining 18 were reported in meeting abstracts. Each case’s summary is presented in Supplementary Table S3. The reports’ methodological quality was graded as 5 in 64 cases, 4 in 13 cases, 3 in 14 cases, and 2 in eight cases. Most cases were reported in the US (n = 32), followed by Australia (n = 15), Canada (n = 9), UK (n = 6), and Japan (n = 6). The number of reported cases has increased since the first case was reported in 2015 (Fig. 2).

### Backgrounds of reported cases

The patients’ mean age was 57 years (Supplementary Fig. S1). Most patients with SAPKA were female (male, 37; female, 55; “undefined”; 7 cases). BMI was reported in only 25 (25.3%) cases, with a median (range) of 38 (16–49) kg/m^2^. SGLT2i was prescribed for treating diabetes in 96 (97.0%) patients, among which 82 had type 2 diabetes, three had type 1 diabetes, and no information was provided regarding the type of diabetes in 11 patients. Dapagliflozin was prescribed for heart failure treatment in a non-diabetes patient [72]. Comorbidities included hypertension (n = 31), coronary artery disease (n = 14), cerebrovascular disease (n = 5), and renal dysfunction (n = 3). Information regarding comorbidities in 40 patients was unavailable. The anesthesia method (n = 91) and medications other than anti-diabetic drugs (n = 81) were not reported in most cases. In 37 (37.4%) cases, baseline HbA1c values were reported, with a mean (standard deviation) of 8.5% (1.5%) (Supplementary Fig. S2). Details regarding preoperative diabetes medication in 75 cases are described in Supplementary Table S4. Postoperative glycosuria was reported in 23 cases; however, the urine glucose levels of the remaining 76 cases were not described. Bariatric surgery was the most common type of surgery (n = 24) in the reported cases, followed by coronary artery bypass grafting (CABG) (n = 18) and cholecystectomy (n = 5) (Supplementary Table S5). Four of the 20 patients undergoing CABG underwent emergency surgery, and 12 underwent elective surgery. Overall, 19 patients underwent emergency surgery and 60 underwent elective surgery. The urgency of the surgery was unknown in the remaining 20 patients. Anesthesia methods were not described in 91 cases. Although general anesthesia tends to be administered in most cases, this was only specified in eight cases. The preoperative fasting period was described in 20 cases (1 day; 11 cases, 3 days; one case, and 5–16 h; eight cases). Perioperative fluid management details have not been described in most cases (Supplementary Table S6).

### Management of SGLT2i

The SGLT2is prescribed in the reported cases were canagliflozin, empagliflozin, dapagliflozin, and “undefined” in 42, 32, 16, and 9 cases, respectively. The treatment duration before the event was not reported in 72 cases. In the remaining 27 patients, the medication duration varied between 1 month and 6 years. The duration of preoperative SGLT2i cessation was reported for 58 patients (58.6%) (Supplemental Table S7). SGLT2i use was ceased 1 day (n = 33), 2 days (n = 9), 18 h (n = 1) or 42 h (n = 1) preoperatively or not ceased at all (n = 14). SGLT2i was omitted perioperatively in one case; however, the duration of preoperative cessation was unclear. There were no preoperative SGLT2i cessation descriptions in the other 40 (40.4%) cases. In all reportedly available cases, SGLT2i administration was stopped in < 3 days before surgery. Among the 19 patients who underwent emergency surgery, four, five, four, and six patients took SGLT2is on the day of surgery, 1 day preoperatively, 2 days preoperatively, or “undefined,” respectively. Postoperatively, SGLT2i was resumed or continued in 15 cases, discontinued in 26, and not reported in 58 cases.

### Characteristics of SAPKA

Table 1 shows a summary of the characteristics of ketoacidosis. The time to diagnose ketoacidosis was reported for 86 (86.9%) patients. Among them, ketoacidosis was diagnosed within 3 days in 57 (66.3%) patients, 10 days in 74 (86.0%), and 14 days in 77 (89.5%) postoperatively. Ketoacidosis was diagnosed intraoperatively in two cases. Ketoacidosis was identified based on laboratory data in 29 cases, whereas 60 presented with symptoms such as nausea, vomiting, respiratory symptoms, and tachycardia. The basis for identifying ketoacidosis was unclear in 10 patients. Regarding blood samples, β-hydroxybutyrate was measured in 45 cases and ketones in 23. The pH value during the diagnosis of ketoacidosis was reported for 93 (93.9%) cases; conversely, the evidence of acidosis was unclear in the remaining 6. HCO_3_, PaCO_2_, and AG values were reported in 87 (87.9%), 54 (54.5%), and 70 (70.7%) cases, respectively. Details of these values are presented in Table 1 and Supplementary Fig. S3. Ketone positivity was detected in urine samples (22 cases), blood samples (43 cases), and both (32 cases). The ketone measurement method was unclear in two cases.

**Table 1:** Characteristics of SAPKA

Category	Details	Number of cases or laboratory data
Time to diagnosis^a^	Intraoperative	2 (2.0%)
	On the day of surgery	11 (11.1%)
	POD 1	25 (25.3%)
	POD 2	12 (12.1%)
	POD 3	9 (9.1%)
	POD4‒7	12 (12.1%)
	POD 8–14	11 (11.1%)
	POD 15–31	3 (4.0%)
	Unclear (within 1 month)	1 (1.0%)
	Not reported	13 (13.1%)
Trigger for identification^b^	Laboratory data	29
	Abdominal pain	10
	Breath shortness/dyspnea	20
	Dizziness	2
	Fatigue, malaise	14
	Fever	2
	Hypertension	2
	Hypotension	3
	Impairment of consciousness	11
	Nausea and/or vomiting	21
	Polyuria	8
	Tachycardia	16
	Tachypnea	15
	Not reported	10
Blood ketones, median [range]	BHB, mmol/L (n = 40)*	5.8 [0.8–62.4]
	Ketones, mmol/L (n = 20)**	4.4 [1.8–12.4]
BGA at the time of diagnosis, median [range]	pH (n = 93)	7.16 [6.82–7.29]
	PaCO_2_, mmHg (n = 55)	22.8 [14.0–35.0]
	HCO_3_, mEq/L (n = 84)	9.0 [2.0–23.0]
	AG, mEq/L (n = 67)	23.0 [6.0–37.0]

*AG* anion gap, *BGA* blood gas analysis, *BHB* β-hydroxybutyrate, *POD* postoperative day, *SAPKA* SGLT2i-associated perioperative ketoacidosis, *SGLT2i* sodium-glucose cotransporter 2 inhibitor. ^a^Number (percentage)

^b^Number

^*^Normal range: lower than 0.4–0.5 mmol/L; **Normal range: lower than 0.6 mmol/L

Blood glucose levels at diagnosis were reported in 93 cases (93.9%); however, the specific value was unclear in 13 cases because it was reported as a range. In the remaining 80 patients, the median [range] blood glucose level during diagnosis was 179 [52–498] mg/dL (Supplementary Fig. S4). In eight cases (10%), the blood glucose level was normal (≤ 125 mg/dL). Sixty-nine (86.2%) patients were hyperglycemic (> 125 mg/dL); however, most patients (59/69, 85.5%) had mild hyperglycemia (≤ 250 mg/dL). Hypoglycemia (blood glucose level, 52 mg/dL) was detected in one non-diabetes patient who received dapagliflozin for heart failure treatment. The most frequently cited precipitating factor were fasting or reduced oral intake (n = 27), followed by surgery or surgical stress (n = 24) and inadequate insulin dose (n = 4).

### Treatment and outcome

Treatment details for ketoacidosis, including fluid, glucose, insulin, and bicarbonate administration, were described in 90 cases. In three cases, the “DKA protocol” was applied to treat ketoacidosis. Most patients (79/99 cases, 79.8%) recovered from ketoacidosis, 40 required ICU admission, seven required mechanical ventilation, and three required dialysis. One patient died from stroke after Moyamoya revascularization surgery. Outcomes were not reported in 19 cases.

## Discussion

In this systematic review of case reports on SAPKA, no SAPKA cases with > 2-day preoperative cessation periods were found, suggesting that a longer cessation period may reduce the risk of developing SAPKA. Although the withholding period was not reported in approximately 40% of cases, this finding is important, as no studies have validated the updated recommendation of preoperative SGLT2i cessation. However, the optimal withholding period remains uncertain. Some patients presented with persistent postoperative glycosuria lasting 3 days or longer despite SGLT2i withdrawal [22, 32, 45, 73], suggesting that SGLT2i has residual efficacy. In this study, approximately 60% of SAPKA occurred within 3 days postoperatively. However, 27.2% developed later than 3 days up to 28 days postoperatively. The specific period of time after discontinuing the medication in which disease onset occurs is unclear. Since in most cases postoperative urine glucose data were not provided (72/99), future studies should present the cessation duration of SGLT2is as well as information regarding perioperative urine glucose to estimate the optimal preoperative withholding period.

As we expected, the number of SAPKA case reports has increased steeply, even after the SGLT2i package insert was revised in 2020. This is likely due to the rapid increase in SGLT2i use as well as increased awareness of SAPKA. Because SAPKA may be easily overlooked, there is an urgent need to elucidate its risk factors. We have made some discoveries in this regard. First, we present evidence that emergency surgery is a risk factor for SAPKA. Here, emergency surgery accounted for a quarter of SAPKA cases. Notably, among 19 patients who underwent emergency surgery, at least nine patients did not take SGLT2i on the day of surgery, indicating that taking SGLT2i on the day of surgery was not the only cause of the SAPKA. Other factors, such as decreased oral intake, dehydration, and infection in these populations, may increase the risk of SAPKA. Second, SAPKA was predominantly observed in female patients. Sex differences in the frequency of SGLT2i-associated ketoacidosis have not been reported to the best of our knowledge. However, a previous study of ketoacidosis associated with canagliflozin suggested that patients prone to increase ketone bodies tended to have advanced type 2 diabetes mellitus (DM) [88]. Thus, the results of the current study, in which SAPKA was predominantly observed in women, might be affected by the baseline severity of DM. Unfortunately, baseline HbA1c values were reported only in 37.4% of cases. Further studies are needed to determine the effect of the severity of DM and sex on the incidence of SAPKA.

In terms of surgery type, bariatric surgery was the most common, which is consistent with the previous study results [11]. Although the effect of obesity on the incidence of SAPKA was unclear, as BMI was reported only in 25.3% of cases, considering that obese patients are prone to ketosis with increased lipolysis, and bariatric surgery is quite common worldwide, the results of our study clearly indicated that bariatric surgery is one of the most important risk factors for SAPKA. Except for bariatric surgery, more invasive surgeries and background infections pose a higher risk of SAPKA.

As mentioned above, most case reports lack important information, other information should be reported. First is a detailed description of perioperative fluid management and glucose and insulin administration. Although there is limited evidence for recommending optimal fluid management for diabetes patients undergoing surgery [89], inappropriate fluid and glucose management can increase the risk of SAPKA [90–92]. Second, the anesthetic method has not been specified in most cases. Perioperative stress response and blood glucose level can be affected by the anesthesia type [93, 94]. Therefore, future studies should include these data.

Some SGLT2is have been indicated for the treatment of heart failure and renal failure in 2020 and 2021, respectively. We found only one case of SAPKA in patients prescribed SGLT2i for non-diabetic indication. In this case, dapagliflozin was prescribed for the treatment of heart failure and continued till the day of surgery. The patient developed SAPKA secondary to postoperative hypoglycemia despite the administration of 1% glucose during surgery. Previous studies have suggested that SGLT2is do not increase the risk of hypoglycemia when compared with placebos outside the perioperative setting [95, 96]. However, this case suggested that SAPKA can occur in non-diabetes patients treated with SGLT2i. Unlike diabetic indication, blood glucose may not be routinely measured in non-diabetes patients, and the optimal withholding duration in non-diabetes patients treated with SGLT2is for heart failure is unknown, as discontinuing the drug may be deleterious to heart failure management. Clinicians should be aware of the risk of SGLT-2-associated hypoglycemia and ketoacidosis in perioperative settings in non-diabetes patients taking these drugs for heart failure.

In most cases, ketoacidosis was not immediately diagnosed, but most patients recovered without complications, indicating that prompt diagnosis and appropriate treatment are correlated with good/improved prognoses.

### Limitations

First, to evaluate preoperative SGLT2i-cessation recommendations and the risk of developing ketoacidosis, it would be necessary to include SGLT2i-treated patients who did not develop ketoacidosis. The exclusion of these patients would make it difficult to completely evaluate SGLT2i-cessation guidelines and would cause a likelihood of biases, such as reporting and selection biases. Second, the duration of preoperative cessation of SGLT2is was reported in only 59 (< 60%) patients. Although all of these patients stopped their SGLT2i < 3 days before surgery, it is difficult to conclusively assess the validity of the updated guidelines. We are currently conducting a multicenter observational study to estimate the incidence and precipitating factors of SAPKA [97]. In the study, the duration of preoperative SGLT2i cessation varied among patients and facilities because the recommendation was changed after the study was initiated, which could help determine the validity of the new recommendation. Third, we found only one case report describing SAPKA in a non-diabetes patient. However, this does not mean that the non-diabetic indication of the SGLT2i does not occur, as their prescriptions are expected to increase rapidly given their recognition as an exciting new treatment option for patients with kidney disease and heart failure [98]. Fourth, we excluded cases in which blood pH was > 7.30, even if blood or urine ketone bodies were elevated. This may exclude cases in which ketosis developed due to SGLT2is. However, ketosis is sometimes physiological and non-fatal. Although a previous systematic review included some ketosis cases, we described a robust SAPKA feature by including only cases that fulfilled the criteria for ketoacidosis.

In conclusion, this study suggests that SGLT2i cessation at least 3 days preoperatively can effectively prevent SAPKA. The steep increase in SAPKA case reports indicates that SAPKA is no longer a rare complication among diabetes patients on SGLT2i medications. However, this study could not identify risk factors for SAPKA, highlighting the need for large prospective epidemiologic studies to identify risk factors. Moreover, high-level vigilance, including postoperative ketone screening, may be recommended for high-risk populations.

## Supplementary Information

Below is the link to the electronic supplementary material.

Supplementary file1 (DOCX 4206 KB)

## Data Availability

The datasets generated during and/or analyzed during the current study are available from the corresponding author on reasonable request.
