# Supplementary material for: Sodium-glucose cotransporter 2 inhibitor-associated perioperative ketoacidosis: a systematic review of case reports
Source: J Anesth. 2023 Feb 27;37(3):465–73. doi: 10.1007/s00540-023-03174-8 (PMC10229478; doi:10.1007/s00540-023-03174-8)
Supplement: Supplementary file 1 — Supplementary file1 (DOCX 4206 KB) [file 540_2023_3174_MOESM1_ESM.docx]

**Sodium-glucose cotransporter 2 inhibitor-associated perioperative ketoacidosis: A systematic review of case reports**

**Supplemental digital contents**

Hiroyuki Seki, M.D., Ph.D.^1^, Satoshi Ideno, M.D., Ph.D.^2^, Toshiya Shiga, M.D., Ph.D.^3^, Hidenobu Watanabe, M.D. ^1^, Motoaki Ono, M.D. ^1^, Akira Motoyasu, M.D. ^1^, Hikari Noguchi, M.D.^1^, Kazuya Kondo, M.D.^1^, Takahiro Yoshikawa, M.D.^1^, Hiroshi Hoshijima, D.D.S., Ph.D.^4^, Shunsuke Hyuga, M.D., Ph.D.^5^, Miho Shishii, M.D.^5^, Ai Nagai, M.D.^5^, Midoriko Higashi, M.D., Ph.D.^6^, Takashi Ouchi, M.D., Ph.D.^7^, Kazuki Yasuda, M.D., Ph.D.^8^, Norifumi Kuratani, M.D., Ph.D., M.P.H.^9^

^1^Department of Anesthesiology, Kyorin University School of Medicine, Tokyo, Japan

^2^Department of Anesthesiology, Kawasaki Municipal Hospital, Kanagawa, Japan

^3^Department of Anesthesiology, International University of Health and Welfare, School of Medicine, Chiba, Japan

^4^Division of Dento-oral Anesthesiology, Tohoku University Graduate School of Dentistry, Miyagi, Japan

^5^Department of Anesthesiology, Kitasato University School of Medicine, Kanagawa, Japan

^6^Department of Anesthesiology and Critical Care Medicine, Graduate School of Medical Sciences, Kyushu University, Fukuoka, Japan

^7^Department of Anesthesiology, Tokyo Dental College Ichikawa General Hospital, Chiba, Japan

^8^ Department of Diabetes, Endocrinology and Metabolism, Kyorin University School of Medicine, Tokyo, Japan

^9^Department of Anesthesia, Saitama Children’s Medical Center, Saitama, Japan

* Corresponding Author: Hiroyuki Seki

Mailing address: 6-20-2 Shinkawa Mitaka, Tokyo 181-8611, Japan

Phone number: 81-422-47-5511

E-mail address: [hshiroyukiseki@gmail.com](mailto:hshiroyukiseki@gmail.com)

**Table of contents**

**1. Supplemental Tables**

- Supplemental Table S1. Search strategies for each database
- Supplemental Table S2. Tool for assessment of methodological quality of the cases
- Supplemental Table S3. Summary of each case
- Supplemental Table S4. Details of preoperative diabetes medication
- Supplemental Table S5. Type of surgery in reported cases
- Supplemental Table S6. Details of perioperative fluid management
- Supplemental Table S7. Preoperative withholding period of the SGLT2is

**2. Supplemental Figures**

- Supplemental Figure S1. Distribution of age in reported cases
- Supplemental Figure S2. Distribution of baseline HbA1c value in reported cases
- Supplemental Figure S3. Details of ketoacidosis
- Supplemental Figure S4. Distribution of blood glucose level at the time of diagnosis

**Supplemental Table S1.** Search strategies for each database

| Data bases | Search strategies |
| --- | --- |
| PubMed | ("SGLT2 inhibitors"[All Fields] OR "sodium-glucose cotransporter 2 inhibitors"[All Fields] OR ("canagliflozin"[MeSH Terms] OR "canagliflozin"[All Fields]) OR ("dapagliflozin"[Supplemental Concept] OR "dapagliflozin"[All Fields] OR "dapagliflozin s"[All Fields]) OR ("empagliflozin"[Supplemental Concept] OR "empagliflozin"[All Fields]) OR ("ertugliflozin"[Supplemental Concept] OR "ertugliflozin"[All Fields]) OR ("ipragliflozin"[Supplemental Concept] OR "ipragliflozin"[All Fields]) OR ("1 5 anhydro 1 5 4 ethoxybenzyl 2 methoxy 4 methylphenyl 1 thioglucitol"[Supplemental Concept] OR "1 5 anhydro 1 5 4 ethoxybenzyl 2 methoxy 4 methylphenyl 1 thioglucitol"[All Fields] OR "luseogliflozin"[All Fields]) OR ("2s 3r 4r 5s 6r 2 4 chloro 3 4 ethoxybenzyl phenyl 6 methylthio tetrahydro 2h pyran 3 4 5 triol"[Supplemental Concept] OR "2s 3r 4r 5s 6r 2 4 chloro 3 4 ethoxybenzyl phenyl 6 methylthio tetrahydro 2h pyran 3 4 5 triol"[All Fields] OR "sotagliflozin"[All Fields]) OR ("6 4 ethylphenyl methyl 3 4 5 6 tetrahydro 6 hydroxymethyl spiro isobenzofuran 1 3h 2 2h pyran 3 4 5 triol"[Supplemental Concept] OR "6 4 ethylphenyl methyl 3 4 5 6 tetrahydro 6 hydroxymethyl spiro isobenzofuran 1 3h 2 2h pyran 3 4 5 triol"[All Fields] OR "tofogliflozin"[All Fields])) AND ("ketosis"[MeSH Terms] OR "ketosis"[All Fields] OR "ketoacidosis"[All Fields] OR ("acidosis"[MeSH Terms] OR "acidosis"[All Fields] OR "acidoses"[All Fields])) |
| Web of Science | (“SGLT2 inhibitors” OR “sodium-glucose cotransporter 2 inhibitors” OR canagliflozin OR dapagliflozin OR empagliflozin OR ertugliflozin OR ipragliflozin OR luseogliflozin OR sotagliflozin OR tofogliflozin) AND (ketoacidosis OR acidosis) |
| EMBASE | (“SGLT2 inhibitors” OR “sodium-glucose cotransporter 2 inhibitors” OR canagliflozin OR dapagliflozin OR empagliflozin OR ertugliflozin OR ipragliflozin OR luseogliflozin OR sotagliflozin OR tofogliflozin) AND (ketoacidosis OR acidosis) |

**Supplemental Table S2.** Tool for assessment of methodological quality of the cases

| Item | Point |
| --- | --- |
| (i) Did the patient represent the whole experience of the investigation? | 1; if yes, 0; if no |
| (ii) Was the diagnosis correctly made? | 1; if yes, 0; if no |
| (iii) Were other important diagnosis excluded | 1; if yes, 0; if no |
| (iv) Were all important data cited in the report? | 1; if yes, 0; if no |
| (v) Was the outcome correctly ascertained? | 1; if yes, 0; if no |
| Total | 0-5 |

**Supplemental Table S3.** Summary of each case

|  | 1^12^ | 2^13^ | 3^14^ | 4^15^ |
| --- | --- | --- | --- | --- |
| Author, year | Abu-Amer N, 2019 | Aggrawal A, 2020 | Alabdaljabar MS, 2021 | Amianda EA, 2021 |
| Country | Israel | USA | Saudi Arabia | USA |
| Type of publication | Scientific paper | Scientific paper | Scientific paper | Scientific paper |
| Age, sex, BMI (kg/m^2^) | 58, F, NA | 53, M, NA | 52, M, NA | 47, F, 40 |
| Type of DM | T2 | T2 | T2 | T2 |
| HbA1c (%) | NA | NA | 10.2 | 8.9 |
| SGLT2i | Canagliflozin | Canagliflozin | Empagliflozin | Canagliflozin |
| Other anti-diabetic drugs | NA | BG | BG, TZD, DPP4i | NA |
| Purpose of SGLT2i | DM | DM | DM | DM |
| Duration of SGLT2i treatment | NA | NA | NA | NA |
| Comorbidities | HTN | HTN | HTN, CAD | HTN |
| Surgery | Laparoscopic gastric wedge resection for gastric perforation | Laparoscopic appendectomy | CABG | Laparoscopic Roux-en-Y gastric bypass for obesity |
| Urgency | Emergency | NA | Elective | Elective |
| Anesthesia | NA | NA | NA | NA |
| Preop fasting | NA | NA | 8h preop | NA |
| Preop insulin, glucose | NA | NA | NA | NA |
| Intraop insulin, glucose | NA | NA | NA | NA |
| Postop insulin, glucose | NA | NA | NA | NA |
| Preop SGLT2i cessation | 2d | NA | 1d | 1d |
| Postop SGLT2i restart | NA | NA | NA | NA |
| Time to presentation | 10d postop | 2d postop | 0d (6h postop) | 7d postop |
| Trigger of diagnosis | Nausea, vomiting, abdominal pain | Breath shortness/dyspnea, tachypnea, tachycardia, abdominal pain, fever | Laboratory data | Breath shortness/dyspnea, tachycardia |
| pH,  HCO_3_ (mEq/L),  AG (mEq/L),  PCO_2_ (mmHg) | 7.29  7  30  15.5 | 7.21  17  20.8  NA | 7.225  14.6  17.4  34.3 | 7.119  5.2  30  16 |
| Urine ketones: UK, Blood ketones: BK | UK: ++++, >4000 mg/dl  BK: positive | UK: positive  BK: BHB 2.69 mmol/L | UK: +++  BK: NA | UK: positive  BK: BHB>4 mmol/L |
| Blood glucose: BG  Urine glucose: UG | BG: NA  UG: NA | BG: 126 mg/dL  UG: NA | BG: 145-188 mg/dL  UG: ++++ | BG: 136 mg/dL  UG: NA |
| Treatment | Glucose, Fluid, Insulin | Glucose, Fluid, Insulin | Glucose, Fluid, Insulin | Glucose, Fluid, HCO_3_, Insulin |
| Outcome | Recovered | ICU admission  Recovered | Recovered | Recovered |
| Precipitating factors | Lack of carbohydrate intake | Preoperative fasting | Preoperative fasting and surgical stress | Perioperative poor oral intake |
| Methodological quality | 5 | 5 | 5 | 5 |

AG, anion gap; BG, biguanides; BHB, β-hydroxybutyrate; CABG, coronary artery bypass grafting; CAD, coronary artery disease; DM, diabetes mellitus; DPP4i, dipeptidyl peptidase-4 inhibitor; HTN, hypertension; NA, not available; SGLT2i, sodium-glucose cotransporter 2 inhibitor; TZD, thiazolidines

**Supplemental Table S3** Summary of each case (continued)

|  | 5^15^ | 6^16^ | 7^17^ | 8^18^ |
| --- | --- | --- | --- | --- |
| Author, year | Amianda EA, 2021 | Andalib A, 2016 | Bnakh I, 2019 | Bobart SA, 2016 |
| Country | USA | Canada | Australia | USA |
| Type of publication | Scientific paper | Scientific paper | Scientific paper | Scientific paper |
| Age, sex, BMI (kg/m^2^) | 54, M, 38 | 58, M, 49 | 64, F, NA | Mid-40s, F, 24 |
| Type of DM | T2 | T2 | T2 | T2 |
| HbA1c (%) | 10 | NA | NA | 6.6 |
| SGLT2i | Empagliflozin | Canagliflozin | Dapagliflozin | Canagliflozin |
| Other anti-diabetic drugs | BG, GLP1RAs | Insulin, BG | Insulin, BG | BG, GLP1RAs |
| Purpose of SGLT2i | DM | DM | DM | DM |
| Duration of SGLT2i treatment | NA | NA | NA | NA |
| Comorbidities | HTN | NA | HTN | NA |
| Surgery | Laparoscopic sleeve gastrectomy for obesity | Sleeve gastrectomy for obesity | Sleeve gastrectomy for obesity | Cosmetic surgery |
| Urgency | Elective | Elective | Elective | NA |
| Anesthesia | NA | NA | NA | NA |
| Preop fasting | NA | NA | NA | NA |
| Preop insulin, glucose | NA | NA | NA | NA |
| Intraop insulin, glucose | NA | NA | NA | NA |
| Postop insulin, glucose | NA | NA | NA | NA |
| Preop SGLT2i cessation | 1d | NA | NA | None |
| Postop SGLT2i restart | NA | NA | NA | No |
| Time to presentation | 8d postop | 9d postop | 28d postop | 2d postop |
| Trigger of diagnosis | Breath shortness/dyspnea, fatigue, malaise | Nausea, vomiting, abdominal pain | Breath shortness/dyspnea, tachypnea, tachycardia, vomiting | Dyspnea, nausea, vomiting |
| pH,  HCO_3_ (mEq/L),  AG (mEq/L),  PCO_2_ (mmHg) | 7.178  6.1  32  16.7 | 7.2  9.4  26  NA | 6.93  2  NA  9 | 7  6  33  14.3 |
| Urine ketones: UK, Blood ketones: BK | UK: NA  BK: BHB 7.4 mmol/L | UK: positive  BK: BHB 10.5 mmol/L | UK: NA  BK: 6.9 mmol/L | UK: NA  BK: BHB 4.3 mmol/L |
| Blood glucose: BG  Urine glucose: UG | BG: 216 mg/dL  UG: NA | BG: 306 mg/dL  UG: NA | BG: 243 mg/dL  UG: NA | BG; 179 mg/dL  UG: NA |
| Treatment | Fluid, insulin | NA | Fluid, glucose, HCO_3_, insulin | Fluid, glucose, HCO_3_, insulin |
| Outcome | Recovered | ICU admission | ICU admission  Recovered | ICU admission  Recovered |
| Precipitating factors | Perioperative poor oral intake | Postoperative lower oral intake and omission of insulin | Dehydration due to reduced oral intake from nausea and vomiting along with reduced insulin doses after surgery | Perioperative poor oral intake |
| Methodological quality | 5 | 3 | 5 |  |

AG, anion gap; BG, biguanides; BHB, β-hydroxybutyrate; DM, diabetes mellitus; GLP1RAs, glucagon-like peptide-1 receptor agonists; HTN, hypertension; NA, not available; SGLT2i, sodium-glucose cotransporter 2 inhibitor

**Supplemental Table S3.** Summary of each case (continued)

|  | 9^19^ | 10^20^ | 11^21^ | 12^21^ |
| --- | --- | --- | --- | --- |
| Author, year | Bonanni FB, 2016 | Bteich F, 2019 | Chacko B, 2018 | Chacko B, 2018 |
| Country | USA | USA | Australia | Australia |
| Type of publication | Scientific paper | Scientific paper | Scientific paper | Scientific paper |
| Age, sex, BMI (kg/m^2^) | 52, F, 42 | 58, F, NA | 55, M, NA | 66, F, NA |
| Type of DM | T2 | T2 | T2 |  |
| HbA1c (%) | NA | NA | NA | NA |
| SGLT2i | Canagliflozin | Empagliflozin | Empagliflozin | Dapagliflozin |
| Other anti-diabetic drugs | BG | Insulin, BG, SU | BG, DPP4i | BG, DPP4i |
| Purpose of SGLT2i | DM | DM | DM | DM |
| Duration of SGLT2i treatment | NA | NA | 6y | Several years |
| Comorbidities | Liver dysfunction | HTN | HTN, CAD | HTN, CAD |
| Surgery | Laparoscopic Roux-en-Y gastric bypass for obesity | VP shunt exchange | Total knee replacement | CABG |
| Urgency | Elective | Emergency | Elective | Elective |
| Anesthesia | NA | NA | NA | NA |
| Preop fasting | 1d preop |  | NA | NA |
| Preop insulin, glucose | NA | NA | NA | NA |
| Intraop insulin, glucose | NA | NA | NA | NA |
| Postop insulin, glucose | NA | NA | NA | Postoperative insulin |
| Preop SGLT2i cessation | None | 2d | None | 1d |
| Postop SGLT2i restart | NA | NA | Not discontinued | NA |
| Time to presentation | 1d postop | 1d postop | 6d postop | 1d (20h) postop |
| Trigger of diagnosis | Polyuria | Laboratory data | Laboratory data | Laboratory data  Polyuria |
| pH,  HCO_3_ (mEq/L),  AG (mEq/L),  PCO_2_ (mmHg) | 7.06  5.5  18  19.9 | 7.2  NA  >28 mEq/L  < 5 mmHg | 7.08  6.5  20  10 | 7.26  13.3  NA  30.2 |
| Urine ketones: UK, Blood ketones: BK | UK: 7.7 mmol/L  BK: 500 mmol/L | UK: > 7.7 mmol/L  BK: BHB 10.1 mmol/L | UK: NA  BK: 2 mmol/L | UK: NA  BK: 1.8 mmol/L |
| Blood glucose: BG  Urine glucose: UG | BG: 112 mg/dL  UG: NA | BG: 143 mg/dL  UG: >1000 mg/dL | BG: 180 mg/dL  UG: NA | BG: 165.6 mg/dL  UG: NA |
| Treatment | Fluid, glucose, HCO_3_, insulin | Fluid, glucose, insulin | Fluid, glucose, HCO_3_, insulin | Fluid, glucose, insulin |
| Outcome | ICU admission  Recovered | Recovered | ICU Admission  Recovered | Mechanical ventilation  Recovered |
| Precipitating factors | Metformin, preoperative ketogenic diet, surgical stress | Surgical stress and inadequate calorie intake | Surgical stress, continuation of anti-diabetic drugs | Surgical stress, weaning of the insulin/dextrose infusion |
| Methodological quality | 5 | 5 | 5 | 5 |

AG, anion gap; BG, biguanides; BHB, β-hydroxybutyrate; CABG, coronary artery bypass grafting; CAD, coronary artery disease; DM, diabetes mellitus; DPP4i, dipeptidyl peptidase-4 inhibitor; HTN, hypertension; NA, not available; SGLT2i, sodium-glucose cotransporter 2 inhibitor; SU, sulfonyl urea; VP, ventriculoperitoneal

**Supplemental Table S3.** Summary of each case (continued)

|  | 13^22^ | 14^23^ | 15^24^ | 16^25^ |
| --- | --- | --- | --- | --- |
| Author, year | Chong ZM, 2018 | Chow YY, 2016 | Curanaj FM, 2020 | Darwish AM, 2019 |
| Country | UK | Australia | USA | USA |
| Type of publication | Meeting abstract | Scientific paper | Meeting abstract | Scientific paper |
| Age, sex, BMI (kg/m^2^) | 52, F, NA | 48, F, NA | 29, F, NA | 38, M, NA |
| Type of DM | T2 | T2 | T2 | T2 |
| HbA1c (%) | 7.2 | NA | NA | NA |
| SGLT2i | Canagliflozin | Canagliflozin | Canagliflozin | Canagliflozin |
| Other anti-diabetic drugs | BG, SU | Insulin, BG | GLP1RAs | BG |
| Purpose of SGLT2i | DM | DM | DM | DM |
| Duration of SGLT2i treatment | NA | 12mo | NA | NA |
| Comorbidities | None | CVD | None | None |
| Surgery | Dental extraction | Cerebral aneurysm clipping | Sleeve gastrectomy for obesity | Laparoscopic sleeve gastrectomy for obesity |
| Urgency | Elective | Elective | Elective | Elective |
| Anesthesia | GA | NA | NA | NA |
| Preop fasting | NA | NA | Restricted to clear liquids for two days | NA |
| Preop insulin, glucose | NA | Reduction in insulin dose | No insulin | NA |
| Intraop insulin, glucose | NA | NA | NA | NA |
| Postop insulin, glucose | NA | NA | No insulin | NA |
| Preop SGLT2i cessation | 1d | 1d | 1d | NA |
| Postop SGLT2i restart | None | 1d postop | NA | 2d postop |
| Time to presentation | 6d postop | 2d postop | 1d postop | 8d postop |
| Trigger of diagnosis | Vomiting | Nausea | Laboratory data | Tachypnea, fatigue, malaise |
| pH,  HCO_3_ (mEq/L),  AG (mEq/L),  PCO_2_ (mmHg) | 7.086  3  NA  NA | 7.14  NA  NA  NA | 7.03  NA  Elevated  <10 mmHg | 6.9  6  35  13 |
| Urine ketones: UK, Blood ketones: BK | UK: NA  BK: 5.1 mmol/L | UK: NA  BK: 3.4 mmol/L | UK: NA  BK: BHB>4.5 mmol/L | UK: large amounts  BK: 4600 mmol/L |
| Blood glucose: BG  Urine glucose: UG | BG: 205.2 mg/dL  UG: positive | BG: 127.8 mg/dL  UG: NA | BG: 120 mg/dL  UG: NA | BG: 136 mg/dL  UG: NA |
| Treatment | DKA care pathway | Fluid, insulin | Fluid, glucose, insulin | Fluid, glucose, HCO_3_, insulin |
| Outcome | Recovered | Recovered | ICU admission | ICU admission, mechanical ventilation,  Recovered |
| Precipitating factors | NA | Poor oral intake from nausea | Surgery with concomitant low caloric intake and dehydration | Restricted calorie intake |
| Methodological quality | 5 | 5 | 3 | 5 |

AG, anion gap; BG, biguanides; BHB, β-hydroxybutyrate; CVD, cerebrovascular disease; DM, diabetes mellitus; GA, general anesthesia; GLP1RAs, glucagon-like peptide-1 receptor agonists; NA, not available; SGLT2i, sodium-glucose cotransporter 2 inhibitor; SU, sulfonyl urea

**Supplemental Table S3.** Summary of each case (continued)

|  | 17^25^ | 18^26^ | 19^27^ | 20^27^ |
| --- | --- | --- | --- | --- |
| Author, year | Darwish AM, 2019 | Di Palma R, 2020 | Dizon S, 2017 | Dizon S, 2017 |
| Country | USA | UK | Canada | Canada |
| Type of publication | Scientific paper | Meeting abstract | Scientific paper | Scientific paper |
| Age, sex, BMI (kg/m^2^) | 60, M, NA | 57, M, NA | 55, F, NA |  |
| Type of DM | T2 | T2 | T2 | T2 |
| HbA1c (%) | NA | NA | 9.3 | NA |
| SGLT2i | Canagliflozin | Empagliflozin | Canagliflozin | Canagliflozin |
| Other anti-diabetic drugs | BG | NA | Insulin, BG | BG, DPP4i, SU |
| Purpose of SGLT2i | DM | DM | DM | DM |
| Duration of SGLT2i treatment | NA | NA | NA | NA |
| Comorbidities | None | CAD | None | None |
| Surgery | Small-bowel resection | OPCABG | Roux-en-Y gastric bypass surgery for obesity | Cholecystectomy |
| Urgency | Emergency | Elective | Elective | Elective |
| Anesthesia | NA | GA | NA | NA |
| Preop fasting | NA | NA | NA | NA |
| Preop insulin, glucose | NA | NA | Elective | Elective |
| Intraop insulin, glucose | NA | NA | Elective | Elective |
| Postop insulin, glucose | NA | Postoperative insulin | Postoperative insulin discontinued | Elective |
| Preop SGLT2i cessation | Unclear (Held since the operation) | 1d | NA | NA |
| Postop SGLT2i restart | None | None | 7d postop | NA |
| Time to presentation | 3d postop | 1d postop | 20d postop | 3d postop |
| Trigger of diagnosis | Laboratory data | Laboratory data | Weakness and inability to tolerate oral fluids | Laboratory data |
| pH,  HCO_3_ (mEq/L),  AG (mEq/L),  PCO_2_ (mmHg) | 7.16  7.7  NA  22 | 7.25  15.2  NA  NA | 7.16  10  18  28 | 7.09  4  21  13 |
| Urine ketones: UK, Blood ketones: BK | UK: ++++  BK: NA | UK: ++++  BK: NA | UK: +++  BK: BHB 5.4 mmol/L | UK: +++  BK: BHB 6.6 mmol/L |
| Blood glucose: BG  Urine glucose: UG | BG: NA  UG: ++++ | BG: 108-270 mg/dL  UG: NA | BG: 183.6 mg/dL  UG: NA | BG: 144 mg/dL  UG: NA |
| Treatment | Fluid, insulin | Fluid, insulin | Fluid, insulin | Fluid, insulin |
| Outcome | ICU admission  Recovered | Recovered | Recovered | Recovered |
| Precipitating factors | Postoperative fasting | Surgical stress | Dehydration, ketotic diet | NA |
| Methodological quality | 5 | 5 | 5 | 5 |

AG, anion gap; BG, biguanides; BHB, β-hydroxybutyrate; OPCABG, off-pump coronary artery bypass grafting; CAD, coronary artery disease; DM, diabetes mellitus; DPP4i, dipeptidyl peptidase-4 inhibitor; GA, general anesthesia; NA, not available; SGLT2i, sodium-glucose cotransporter 2 inhibitor; SU, sulfonyl urea

**Supplemental Table S3.** Summary of each case (continued)

|  | 21^27^ | 22^28^ | 23^29^ | 24^30^ |
| --- | --- | --- | --- | --- |
| Author, year | Dizon S, 2017 | Ejaz S, 2016 | Erondu N, 2015 | Evans LC, 2018 |
| Country | Canada | USA | USA | UK |
| Type of publication | Scientific paper | Meeting abstract | Scientific paper | Meeting abstract |
| Age, sex, BMI (kg/m^2^) | 74, F, NA | 66, M, NA | 73, M, 26 | 57, F, NA |
| Type of DM | T2 | T1 | T1 | T2 |
| HbA1c (%) | 7 | NA | 9.1 | NA |
| SGLT2i | Canagliflozin | Detail unclear | Canagliflozin | Dapagliflozin |
| Other anti-diabetic drugs | BG, DPP4i, SU | Insulin, BG, SU | Insulin | NA |
| Purpose of SGLT2i | DM | DM | DM | DM |
| Duration of SGLT2i treatment | NA | Recently started | NA | NA |
| Comorbidities | NA | NA | NA | CAD |
| Surgery | Operative repair of an intertrochanteric fracture | Knee surgery | Laparoscopic cholecystectomy | CABG |
| Urgency | NA | NA | NA | NA |
| Anesthesia | NA | NA | NA | NA |
| Preop fasting | NA | NA | NA | NA |
| Preop insulin, glucose | NA | NA | NA | NA |
| Intraop insulin, glucose | NA | NA | NA | NA |
| Postop insulin, glucose | NA | NA | NA | NA |
| Preop SGLT2i cessation | NA | NA | NA | 1d |
| Postop SGLT2i restart | 1d postop | NA | NA | 1d postop |
| Time to presentation | 5d postop | 3d postop | On the day of surgery | 3d postop |
| Trigger of diagnosis | Fatigue, malaise, confusion | Dyspnea, nausea, vomiting, dizziness | NA | Laboratory data |
| pH,  HCO_3_ (mEq/L),  AG (mEq/L),  PCO_2_ (mmHg) | 7.14  11  21  32 | 7.16  5  35  NA | 7.24  15  6  NA | 7.05  NA  NA  NA |
| Urine ketones: UK, Blood ketones: BK | UK: ++++  BK: BHB 7.3 mmol/L | UK: NA  BK: positive | UK: NA  BK: positive | UK: raised  BK: raised |
| Blood glucose: BG  Urine glucose: UG | BG: 213 mg/dL  UG: NA | BG: 361 mg/dL  UG: NA | BG: 369 mg/dL  UG: NA | BG: 158.4-271.8 mg/dL  UG: NA |
| Treatment | DKA protocol | Fluid, HCO_3_, insulin | NA | Fluid, insulin |
| Outcome | Recovered | ICU admission  Recovered | NA | NA |
| Precipitating factors | NA | Surgery | Surgery | Intercurrent illness |
| Methodological quality | 5 | 3 | 3 | 4 |

AG, anion gap; BG, biguanides; BHB, β-hydroxybutyrate; CABG, coronary artery bypass grafting; CAD, coronary artery disease; DM, diabetes mellitus; DPP4i, dipeptidyl peptidase-4 inhibitor; NA, not available; SGLT2i, sodium-glucose cotransporter 2 inhibitor; SU, sulfonyl urea

**Supplemental Table S3.** Summary of each case (continued)

|  | 25^31^ | 26^32^ | 27^33^ | 28^34^ |
| --- | --- | --- | --- | --- |
| Author, year | Gelaye A, 2016 | Ghofrain H, 2015 | Gomez-Sanchez CM, 2021 | Gonzalez ME, 2017 |
| Country | USA | USA | USA | USA |
| Type of publication | Scientific paper | Meeting abstract | Scientific paper | Meeting abstract |
| Age, sex, BMI (kg/m^2^) | 54, M, NA | 38, M, NA | 73, M, NA | 65, F, 36 |
| Type of DM | NA | T2 | T2 | T2 |
| HbA1c (%) | NA | NA | 8.8 | NA |
| SGLT2i | Canagliflozin | Canagliflozin | Empagliflozin | Canagliflozin |
| Other anti-diabetic drugs | Insulin | NA | Insulin, BG | BG, GLP1RAs |
| Purpose of SGLT2i | DM | DM | DM | DM |
| Duration of SGLT2i treatment | 3yr | 3mo | Recently started | NA |
| Comorbidities | NA | NA | HTN, CAD, Renal dysfunction | HTN |
| Surgery | Laparoscopic appendectomy | Gastric sleeve bypass for obesity | Femoral endarterectomy and femoral to below-knee bypass | Laparoscopic sleeve gastrectomy for obesity |
| Urgency | NA | Elective | Elective | Elective |
| Anesthesia | NA | NA | NA | NA |
| Preop fasting | NA | NA | NA | NA |
| Preop insulin, glucose | NA | NA | NA | NA |
| Intraop insulin, glucose | NA | NA | NA | NA |
| Postop insulin, glucose | NA | NA | Not received insulin on POD1 | NA |
| Preop SGLT2i cessation | NA | NA | 1d | 1d |
| Postop SGLT2i restart | NA | None | NA | NA |
| Time to presentation | 2d postop | 10d postop | 2d postop | 1d postop |
| Trigger of diagnosis | Abdominal pain, confusion, chest discomfort, fatigue, | Dyspnea, fatigue | Delirium | Fatigue, nausea |
| pH,  HCO_3_ (mEq/L),  AG (mEq/L),  PCO_2_ (mmHg) | 7.058  9  37  NA | 6.9  5  37  13 | 7.2  8.9  24  19 | 7.1  NA  17  20.8 |
| Urine ketones: UK, Blood ketones: BK | UK: NA  BK: BHB 12.4 mmol/L | UK: >2.5 mmol/L  BK: | UK: 13.8 mmol/L  BK: NA | UK: NA  BK: BHB 5.1 mmol/L |
| Blood glucose: BG  Urine glucose: UG | BG: 142 mg/dL  UG: NA | BG: 152 mg/dL  UG: >1000 mg/dL | BG: 170-200 mg/dL  UG: NA | BG: 128 mg/dL  UG: NA |
| Treatment | Fluid, glucose, insulin | Fluid, glucose | Fluid, insulin | Fluid, glucose, HCO_3_, insulin |
| Outcome | ICU admission, dialysis, mechanical ventilation,  Recovered | ICU admission, mechanical ventilation,  Recovered | Recovered | Recovered |
| Precipitating factors | Reduced oral intake | Low calorie diet | Surgical stress | Fasting, volume depletion, deficiency of insulin |
| Methodological quality | 5 | 5 | 5 | 5 |

AG, anion gap; BG, biguanides; BHB, β-hydroxybutyrate; CAD, coronary artery disease; DM, diabetes mellitus; GLP1RAs, glucagon-like peptide-1 receptor agonists; HTN, hypertension; NA, not available; SGLT2i, sodium-glucose cotransporter 2 inhibitor

**Supplemental Table S3.** Summary of each case (continued)

|  | 29^35^ | 30^36^ | 31^37^ | 32^37^ |
| --- | --- | --- | --- | --- |
| Author, year | Goto S, 2021 | Hamill CF, 2017 | Hawkins AM, 2017 | Hawkins AM, 2017 |
| Country | Japan | Ireland | Australia | Australia |
| Type of publication | Scientific paper | Meeting abstract | Scientific paper | Scientific paper |
| Age, sex, BMI (kg/m^2^) | 76, F, NA | 42, F, NA | 43, F, NA | 45, F, NA |
| Type of DM | T2 | T2 | T2 | T2 |
| HbA1c (%) | NA | 9.2 | NA | NA |
| SGLT2i | Canagliflozin | Empagliflozin | Empagliflozin | Empagliflozin |
| Other anti-diabetic drugs | BG | BG, GLP1RAs | BG, DPP4i | BG |
| Purpose of SGLT2i | DM | DM | DM | DM |
| Duration of SGLT2i treatment | NA | NA | NA | NA |
| Comorbidities | NA | HTN | HTN | NA |
| Surgery | Gastrojejunal bypass | Abdominoplasty | Laparotomy for hematoma drainage | Cholecystectomy |
| Urgency | Elective | Elective | Emergency | NA |
| Anesthesia | NA | NA | NA | NA |
| Preop fasting | NA | NA | NA | None |
| Preop insulin, glucose | NA | NA | NA | NA |
| Intraop insulin, glucose | NA | NA | NA | NA |
| Postop insulin, glucose | NA | NA | NA | NA |
| Preop SGLT2i cessation | 1d | Unclear (Omitted perioperatively) | None | NA |
| Postop SGLT2i restart | NA | Gradually reinstituted | None | None |
| Time to presentation | 2d postop | 1d postop | On the day of surgery | 1d postop |
| Trigger of diagnosis | Laboratory data | Breath shortness, dyspnea, vomiting | Laboratory data | Tachycardia |
| pH,  HCO_3_ (mEq/L),  AG (mEq/L),  PCO_2_ (mmHg) | 7.27  13.5  23.7  NA | 6.92  NA  25.6  NA | 7.02  5  NA  NA | 7.19  8  NA  NA |
| Urine ketones: UK, Blood ketones: BK | UK: NA  BK: BHB 18.6 mmol/L | UK: NA  BK: 4.4 mmol/L | UK: NA  BK: 3.2 mmol/L | UK: NA  BK: 3.4 mmol/L |
| Blood glucose: BG  Urine glucose: UG | BG: NA  UG: NA | BG: 178.2 mg/dL  UG: NA | BG: 182 mg/dL  UG: NA | BG: 144 mg/dL  UG: NA |
| Treatment | Fluid, glucose, insulin | Fluid, glucose, insulin | Fluid, glucose | Fluid, insulin |
| Outcome | ICU admission  Recovered | Recovered | ICU admission  Recovered | Recovered |
| Precipitating factors | Surgical stress | Prior massive weight loss, surgery, perioperative fasting | NA | NA |
| Methodological quality | 5 | 5 | 5 | 5 |

AG, anion gap; BG, biguanides; BHB, β-hydroxybutyrate; DM, diabetes mellitus; DPP4i, dipeptidyl peptidase-4 inhibitor; GLP1RAs, glucagon-like peptide-1 receptor agonists; HTN, hypertension; NA, not available; SGLT2i, sodium-glucose cotransporter 2 inhibitor

**Supplemental Table S3.** Summary of each case (continued)

|  | 33^38^ | 34^39^ | 35^39^ | 36^40^ |
| --- | --- | --- | --- | --- |
| Author, year | Hoffman C, 2017 | Horikoshi R, 2019 | Horikoshi R, 2019 | Howard J, 2019 |
| Country | USA | Japan | Japan | USA |
| Type of publication | Scientific paper | Meeting abstract | Scientific paper | Scientific paper |
| Age, sex, BMI (kg/m^2^) | 55, F, NA | 48, F, NA | 53, M, NA | 46, F, NA |
| Type of DM | T2 | NA | NA | T2 |
| HbA1c (%) | NA | NA | NA | NA |
| SGLT2i | Canagliflozin | Canagliflozin | Empagliflozin | Empagliflozin |
| Other anti-diabetic drugs | Insulin, BG | NA | NA | Insulin, BG |
| Purpose of SGLT2i | DM | NA | NA | DM |
| Duration of SGLT2i treatment | NA | NA | NA | NA |
| Comorbidities | NA | CAD | CAD | NA |
| Surgery | Reconstructive surgery for breast cancer | CABG | CABG | Debridement for soft tissue infection of the buttocks |
| Urgency | Elective | Emergency | Emergency | Emergency |
| Anesthesia | NA | NA | NA | NA |
| Preop fasting | NA | NA | NA | 3d |
| Preop insulin, glucose | Preoperative insulin | NA | NA | NA |
| Intraop insulin, glucose | NA | NA | NA | NA |
| Postop insulin, glucose | NA | NA | NA | Postoperative insulin |
| Preop SGLT2i cessation | 1d | None | None | NA |
| Postop SGLT2i restart | None | NA | NA | NA |
| Time to presentation | 1d | NA | NA | NA |
| Trigger of diagnosis | Tachycardia, hypotension, confusion, polyuria | Laboratory data | Laboratory data | Laboratory data, breath shortness, dyspnea |
| pH,  HCO_3_ (mEq/L),  AG (mEq/L),  PCO_2_ (mmHg) | 7.08  6  NA  NA | 7.18  8.3  NA  22.8 | 7.29  17.5  NA  37.1 | 7.22  5  NA  12 |
| Urine ketones: UK, Blood ketones: BK | UK: NA  BK: BHB 62.4 mmol/L | UK: +++  BK: NA | UK: +++  BK: NA | UK: NA  BK: BHB 9.4 mmol/L |
| Blood glucose: BG  Urine glucose: UG | BG: 250.2  UG: NA | BG: 144  UG: +++ | BG: 184  UG: ++++ | BG: 115  UG: NA |
| Treatment | Fluid, insulin | Fluid, glucose, insulin | NA | Fluid, glucose, insulin |
| Outcome | Recovered | Recovered | Recovered | Recovered |
| Precipitating factors | Surgical stress | Surgical stress | Surgical stress | NA |
| Methodological quality | 4 | 5 | 5 | 4 |

AG, anion gap; BG, biguanides; BHB, β-hydroxybutyrate; CABG, coronary artery bypass grafting; CAD, coronary artery disease; DM, diabetes mellitus; NA, not available; SGLT2i, sodium-glucose cotransporter 2 inhibitor

**Supplemental Table S3.** Summary of each case (continued)

|  | 37^41^ | 38^41^ | 39^42^ | 40^42^ |
| --- | --- | --- | --- | --- |
| Author, year | Iqbal QZ, 2020 | Iqbal QZ, 2020 | Isaacs M, 2017 | Isaacs M, 2017 |
| Country | USA | USA | Australia | Australia |
| Type of publication | Scientific paper | Scientific paper | Scientific paper | Scientific paper |
| Age, sex, BMI (kg/m^2^) | 56, M, NA | 52, F, NA | 59, M, NA | 64, F, NA |
| Type of DM | T2 | T2 | T2 | T2 |
| HbA1c (%) | NA | NA | NA | NA |
| SGLT2i | Canagliflozin | Canagliflozin | Dapagliflozin | Dapagliflozin |
| Other anti-diabetic drugs | BG | BG | BG | BG, GLP1RAs |
| Purpose of SGLT2i | DM | DM | DM | DM |
| Duration of SGLT2i treatment | A few months | 2y | NA | NA |
| Comorbidities | NA | HTN | None | None |
| Surgery | Roux-en-Y gastric bypass for obesity | Gastric bypass for obesity | Lumber spine fusion | Bilateral total knee replacements |
| Urgency | Elective | NA | Elective | Elective |
| Anesthesia | NA | NA | NA | NA |
| Preop fasting | NA | NA | NA | None |
| Preop insulin, glucose | Preoperative insulin | NA | NA | NA |
| Intraop insulin, glucose | NA | NA | NA | NA |
| Postop insulin, glucose | NA | NA | NA | NA |
| Preop SGLT2i cessation | NA | NA | 1d | 1d |
| Postop SGLT2i restart | None | None | 1d postop | 1d postop |
| Time to presentation | 4d postop | 14d postop | 2d postop | 3d postop |
| Trigger of diagnosis | Laboratory data, breath shortness, dyspnea, tachycardia, abdominal pain, fatigue, polyuria | Breath shortness, dyspnea, fatigue, polyuria | Breath shortness, dyspnea, tachycardia, nausea, vomiting, fever | Reduced level of consciousness |
| pH,  HCO_3_ (mEq/L),  AG (mEq/L),  PCO_2_ (mmHg) | 6.91  4  32  NA | 7.2  8  35  NA | 7.23  10  NA  NA | 7.14  8  NA  NA |
| Urine ketones: UK, Blood ketones: BK | UK: NA  BK: BHB>4.5 mmol/L | UK: ++++  BK: BHB>9 mmol/L | UK: NA  BK: BHB 5.6 mmol/L | UK: NA  BK: BHB 6.1 mmol/L |
| Blood glucose: BG  Urine glucose: UG | BG: 208 mg/dL  UG: | BG: 196 mg/dL  UG: | BG: 122 mg/dL  UG: | BG: 192 mg/dL  UG: |
| Treatment | Fluid, glucose, insulin | Fluid, glucose | Fluid, glucose, insulin | Fluid, glucose, insulin |
| Outcome | ICU admission,  Recovered | ICU admission,  Recovered | Recovered | Recovered |
| Precipitating factors | Bariatric surgery | Bariatric surgery | NA | NA |
| Methodological quality | 5 | 5 | 5 | 5 |

AG, anion gap; BG, biguanides; BHB, β-hydroxybutyrate; DM, diabetes mellitus; GLP1RAs, glucagon-like peptide-1 receptor agonists; HTN, hypertension; NA, not available; SGLT2i, sodium-glucose cotransporter 2 inhibitor

**Supplemental Table S3.** Summary of each case (continued)

|  | 41^43^ | 42^43^ | 43^44^ | 44^45^ |
| --- | --- | --- | --- | --- |
| Author, year | Jhaveri U, 2019 | Jhaveri U, 2019 | Kameda Y, 2019 | Kapila V, 2021 |
| Country | Australia | Australia | Japan | USA |
| Type of publication | Scientific paper | Scientific paper | Scientific paper | Scientific paper |
| Age, sex, BMI (kg/m^2^) | 43, F, NA | 41, F, 41 | 65, F, 26 | 51, F, NA |
| Type of DM | T2 | T2 | T2 | T2 |
| HbA1c (%) | NA | 11.7 | 7.1 | NA |
| SGLT2i | Empagliflozin | Empagliflozin | Empagliflozin | Canagliflozin |
| Other anti-diabetic drugs | Insulin, BG | BG | BG | Insulin, BG |
| Purpose of SGLT2i | DM | DM | DM | DM |
| Duration of SGLT2i treatment | NA | NA | NA | NA |
| Comorbidities | HTN | NA | HTN, CAD | NA |
| Surgery | Laparoscopic cholecystectomy | Debridement | CABG | Laparoscopic sleeve gastrectomy for obesity |
| Urgency | Emergency | NA | Emergency | NA |
| Anesthesia | NA | NA | NA | NA |
| Preop fasting | NA | NA | NA | NA |
| Preop insulin, glucose | Insulin discontinued preoperatively | NA | NA | NA |
| Intraop insulin, glucose | NA | NA | NA | NA |
| Postop insulin, glucose | NA | NA | NA | NA |
| Preop SGLT2i cessation | NA | NA | 1d | NA |
| Postop SGLT2i restart | None | None | None | None |
| Time to presentation | Intraop | Unclear | On the day of surgery | 1d |
| Trigger of diagnosis | Laboratory data | Laboratory data | Laboratory data | Tachycardia, hypertension |
| pH,  HCO_3_ (mEq/L),  AG (mEq/L),  PCO_2_ (mmHg) | 6.82  6  28  NA | 7.29  14  18  NA | 7.22  NA  NA  NA | 7.21  8  37  60 |
| Urine ketones: UK, Blood ketones: BK | UK: NA  BK: ketosis | UK: NA  BK: ketoacidosis | UK: ++++  BK: NA | UK: positive  BK: NA |
| Blood glucose: BG  Urine glucose: UG | BG: 344 mg/dL  UG: NA | BG: 250 mg/dL  UG: NA | BG: 130 mg/dL  UG: NA | BG: 180 mg/dL  UG: positive |
| Treatment | Fluid, insulin | Fluid, insulin | Fluid, HCO_3_ | Fluid, glucose, insulin |
| Outcome | ICU admission | Recovered | Recovered | ICU admission,  Recovered |
| Precipitating factors | Preoperative dehydration due to vomiting | Infection | NA | Ketotic diet |
| Methodological quality | 2 | 2 | 5 | 5 |

AG, anion gap; BG, biguanides; BHB, β-hydroxybutyrate; CABG, coronary artery bypass grafting; CAD, coronary artery disease; DM, diabetes mellitus; HTN, hypertension; NA, not available; SGLT2i, sodium-glucose cotransporter 2 inhibitor

**Supplemental Table S3.** Summary of each case (continued)

|  | 45^46^ | 46^47^ | 47^48^ | 48^49^ |
| --- | --- | --- | --- | --- |
| Author, year | Kitahara C, 2021 | Klosko R, 2021 | Kuchay MS, 2021 | Lane S, 2018 |
| Country | Japan | USA | India | Canada |
| Type of publication | Scientific paper | Scientific paper | Scientific paper | Scientific paper |
| Age, sex, BMI (kg/m^2^) | 59, M, 24 | 61, M, NA | 53, F, 19 | 42, F, 40 |
| Type of DM | T2 | T2 | T2 | T2 |
| HbA1c (%) | 9.4 | 6.6 | 8 | NA |
| SGLT2i | Empagliflozin | Empagliflozin | Empagliflozin | Canagliflozin |
| Other anti-diabetic drugs | Insulin | BG, DPP4i | BG, DPP4i | Insulin, BG, DPP4i |
| Purpose of SGLT2i | DM | DM | DM | DM |
| Duration of SGLT2i treatment | 18mo | NA | 8wk | NA |
| Comorbidities | NA | NA | NA | None |
| Surgery | Thoracoscopic debridement and intrathoracic lavage | CABG | CABG | Laparoscopic Roux-en-Y gastric bypass for obesity |
| Urgency | Elective | Elective | Elective | Elective |
| Anesthesia | NA | GA | NA | NA |
| Preop fasting | 1d | 1d | NA | 1d |
| Preop insulin, glucose | Preoperative insulin without glucose load | NA | NA | Reduced dose of insulin |
| Intraop insulin, glucose | Intraoperative insulin with glucose load | Intraoperative insulin | NA | NA |
| Postop insulin, glucose | Postoperative insulin with glucose load | No postoperative insulin | NA | Postoperative insulin |
| Preop SGLT2i cessation | 2d | 1d | 2d | None |
| Postop SGLT2i restart | NA | NA | NA | NA |
| Time to presentation | Intraop | 1d postop | 1d postop | 3d postop |
| Trigger of diagnosis | Laboratory data | Laboratory data | Laboratory data, altered sensorium | Breath shortness, dyspnea, tachycardia, hypertension, confusion, dizziness, severe thirst |
| pH,  HCO_3_ (mEq/L),  AG (mEq/L),  PCO_2_ (mmHg) | 7.12  16.7  NA  53.6 | Anion-gap metabolic acidosis  14  19  NA | 7.01  10.2  10  NA | 6.88  2.5  28  14 |
| Urine ketones: UK, Blood ketones: BK | UK: +++  BK: 12.4 mmol/L | UK: NA  BK: BHB 5 mmol/L | UK: ++++  BK: NA | UK: NA  BK: NA |
| Blood glucose: BG  Urine glucose: UG | BG: 162 mg/dL  UG: ++++ | BG: NA  UG: NA | BG: 148 mg/dL  UG: NA | BG: 180 mg/dL  UG: NA |
| Treatment | Fluid, glucose, insulin | Fluid, glucose, insulin | Fluid, glucose, insulin | Fluid, glucose, insulin |
| Outcome | Recovered | Recovered | Recovered | ICU admission,  Recovered |
| Precipitating factors | An insufficient dosage of glucose and insulin | NA | Surgical stress | Preoperative low-calorie diet |
| Methodological quality | 5 | 2 | 5 | 5 |

AG, anion gap; BG, biguanides; BHB, β-hydroxybutyrate; CABG, coronary artery bypass grafting; DM, diabetes mellitus; DPP4i, dipeptidyl peptidase-4 inhibitor; GA, general anesthesia; NA, not available; SGLT2i, sodium-glucose cotransporter 2 inhibitor

**Supplemental Table S3.** Summary of each case (continued)

|  | 49^50^ | 50^51^ | 51^52^ | 52^53^ |
| --- | --- | --- | --- | --- |
| Author, year | Lau A, 2018 | Lee D, 2021 | Li J, 2016 | Lindsay PJ, 2020 |
| Country | Canada | UK | Canada | USA |
| Type of publication | Scientific paper | Scientific paper | Meeting abstract | Scientific paper |
| Age, sex, BMI (kg/m^2^) | 54, M, 31 | 70, F, NA | 55, F, NA | 51, M, NA |
| Type of DM | T2 | T2 | T2 | T2 |
| HbA1c (%) | 9.2 | NA | NA | 9 |
| SGLT2i | Empagliflozin | Empagliflozin | Canagliflozin | Empagliflozin |
| Other anti-diabetic drugs | Insulin | NA | Insulin, GLP1RAs | BG |
| Purpose of SGLT2i | DM | DM | DM | DM |
| Duration of SGLT2i treatment | NA | NA | NA | NA |
| Comorbidities | CAD | NA | NA | HTN |
| Surgery | CABG | Appendectomy | Roux-en-Y bypass surgery for obesity | Debridement for Fournier’s gangrene |
| Urgency | Elective | Emergency | Elective | Emergency |
| Anesthesia | NA | NA | NA | GA |
| Preop fasting | NA | NA | NA | NA |
| Preop insulin, glucose | NA | NA | NA | NA |
| Intraop insulin, glucose | Intraop insulin | NA | NA | Intraop insulin and glucose |
| Postop insulin, glucose | Insulin discontinued | NA | Insulin discontinued | NA |
| Preop SGLT2i cessation | 2d | NA | NA | 1d |
| Postop SGLT2i restart | None | NA | 19d postop | NA |
| Time to presentation | 1d postop | 1d postop | 22d postop | Intraop |
| Trigger of diagnosis | Tachypnea | Nausea, vomiting | Laboratory data  Tachycardia, hypotension, fatigue, malaise | Laboratory data |
| pH,  HCO_3_ (mEq/L),  AG (mEq/L),  PCO_2_ (mmHg) | 7.24  14  15  32 | Significant metabolic acidosis | 7.16  10  16  NA | 7.12  NA  31  NA |
| Urine ketones: UK, Blood ketones: BK | UK: NA  BK: BHB 5.3 mmol/L | UK: NA  BK: markedly raised ketones | UK: NA  BK: BHB 5.4 mmol/L | UK: positive  BK: NA |
| Blood glucose: BG  Urine glucose: UG | BG: 216 mg/dL  UG: NA | BG: <198 mg/dL  UG: NA | BG: 183.6 mg/dL  UG: NA | BG: 315 mg/dL  UG: NA |
| Treatment | Fluid, glucose, HCO_3_, insulin | Fluid, insulin | Fluid, glucose, insulin | Fluid, glucose, insulin |
| Outcome | Recovered | Recovered | Recovered | ICU admission  Recovered |
| Precipitating factors | NA | NA | Postoperative insulin reduction | An active infection and decreased oral intake |
| Methodological quality | 5 | 2 | 4 | 5 |

AG, anion gap; BG, biguanides; BHB, β-hydroxybutyrate; CABG, coronary artery bypass grafting; CAD, coronary artery disease; DM, diabetes mellitus; GA, general anesthesia; GLP1RAs, glucagon-like peptide-1 receptor agonists; HTN, hypertension; NA, not available; SGLT2i, sodium-glucose cotransporter 2 inhibitor

**Supplemental Table S3.** Summary of each case (continued)

|  | 53^54^ | 54^55^ | 55^56^ | 56^57^ |
| --- | --- | --- | --- | --- |
| Author, year | Luo X, 2022 | Mackintosh C, 2020 | Malicek D, 2020 | McCabe DE, 2020 |
| Country | China | USA | Germany | USA |
| Type of publication | Scientific paper | Scientific paper | Scientific paper | Scientific paper |
| Age, sex, BMI (kg/m^2^) | 57, F, NA | 68, F, NA | 76, M, NA | 65, F, NA |
| Type of DM | NA | T2 | T2 | T2 |
| HbA1c (%) | NA | NA | NA | NA |
| SGLT2i | Dapagliflozin | Empagliflozin | Dapagliflozin | Canagliflozin |
| Other anti-diabetic drugs | BG | NA | BG | BG, GLP1RAs |
| Purpose of SGLT2i | DM | DM | DM | DM |
| Duration of SGLT2i treatment | NA | NA | NA | NA |
| Comorbidities | HTN, CVD | None | COPD | HTN |
| Surgery | Laparoscopic distal pancreatectomy | Resection of meningioma | Partial pancreatic resection | Laparoscopic sleeve gastrectomy for obesity |
| Urgency | Elective | Elective | Elective | Elective |
| Anesthesia | NA | NA | GA | NA |
| Preop fasting | NA | NA | NA | NA |
| Preop insulin, glucose | NA | NA | NA | NA |
| Intraop insulin, glucose | NA | NA | NA | NA |
| Postop insulin, glucose | NA | NA | NA | NA |
| Preop SGLT2i cessation | NA | 1d | 1d | NA |
| Postop SGLT2i restart | 3d postop | NA | NA | NA |
| Time to presentation | 8d postop | 1d postop | On the day of surgery | 1d postop |
| Trigger of diagnosis | Laboratory data | Confusion | Hypotension | Laboratory data |
| pH,  HCO_3_ (mEq/L),  AG (mEq/L),  PCO_2_ (mmHg) | 7.09  14  36.2  56.7 | 7.2  9  21  NA | 7.185  BE: -12.3  NA  NA | Severely acidotic |
| Urine ketones: UK, Blood ketones: BK | UK: ++++  BK: BHB 10.9 mmol/L | UK: positive  BK: BHB positive | UK: positive  BK: 2.8 mmol/L | UK: +++  BK: BHB 5.1 mmol/L |
| Blood glucose: BG  Urine glucose: UG | BG: 270 mg/dL  UG: NA | BG: 140-160 mg/dL  UG: positive | BG: 240 mg/dL  UG: positive | BG: 128 mg/dL  UG: NA |
| Treatment | Fluid, insulin | Fluid, insulin | Fluid, HCO_3_, insulin | Fluid, insulin |
| Outcome | ICU admission  Recovered | Recovered | ICU admission, dialysis, recovered | Recovered |
| Precipitating factors | Surgery | Perioperative fasting and surgery | NA | Preoperative fasting and surgical stress |
| Methodological quality | 5 | 5 | 4 | 2 |

AG, anion gap; BG, biguanides; BHB, β-hydroxybutyrate; CVD, cerebrovascular disease; COPD, chronic obstructive pulmonary diseases; DM, diabetes mellitus; GLP1RAs, glucagon-like peptide-1 receptor agonists; HTN, hypertension; NA, not available; SGLT2i, sodium-glucose cotransporter 2 inhibitor

**Supplemental Table S3.** Summary of each case (continued)

|  | 57^58^ | 58^59^ | 59^60^ | 60^61^ |
| --- | --- | --- | --- | --- |
| Author, year | McGuiness M, 2021 | Mendonca FM, 2020 | Menhem M, 2017 | Miline L, 2019 |
| Country | USA | Portugal | USA | UK |
| Type of publication | Meeting abstract | Scientific paper | Meeting abstract | Meeting abstract |
| Age, sex, BMI (kg/m^2^) | 71, M, NA | 60, F, 41 | 65, F, 38 | 64, F, NA |
| Type of DM | T2 | T2 | T2 | T2 |
| HbA1c (%) | NA | 8.5 | 7.9 | NA |
| SGLT2i | Unclear | Dapagliflozin | Canagliflozin | Canagliflozin |
| Other anti-diabetic drugs | NA | Insulin, BG | BG, GLP1RAs | BG, SU |
| Purpose of SGLT2i | DM | DM | DM | DM |
| Duration of SGLT2i treatment | NA | NA | NA | NA |
| Comorbidities | None | HTN | None | None |
| Surgery | Total knee arthroplasty | Roux-en-Y gastric bypass for obesity | Laparoscopic sleeve gastrectomy for obesity | Gynecological laparotomy |
| Urgency | Elective | Elective | Elective | Elective |
| Anesthesia | NA | NA | NA | NA |
| Preop fasting | NA | NA | 1d | NA |
| Preop insulin, glucose | NA | NA | NA | NA |
| Intraop insulin, glucose | NA | NA | NA | NA |
| Postop insulin, glucose | NA | Postoperative insulin discontinued | NA | NA |
| Preop SGLT2i cessation | NA | NA | 1d | NA |
| Postop SGLT2i restart | NA | NA | None | NA |
| Time to presentation | 2d postop | 13d postop | 1d postop | 5d postop |
| Trigger of diagnosis | Tachypnea, tachycardia | Fatigue, malaise, altered state of consciousness, dizziness | Laboratory data | Tachypnea, tachycardia, nausea, vomiting, confusion, drowsiness |
| pH,  HCO_3_ (mEq/L),  AG (mEq/L),  PCO_2_ (mmHg) | High anion gap metabolic acidosis  NA  NA  NA | 7  2  31  7.5 | 7.13  <5  19  NA | 7.06  3.4  27.5  1.6 |
| Urine ketones: UK, Blood ketones: BK | UK: positive  BK: BHB positive | UK: positive  BK: NA | UK: NA  BK: BHB 5.9 mmol/L | UK: positive  BK: 3.3 mmol/L |
| Blood glucose: BG  Urine glucose: UG | BG: NA  UG: NA | BG: 498  UG: positive | BG: 128  UG: NA | BG: 140.4  UG: positive |
| Treatment | Fluid, insulin | Fluid, insulin | Fluid, glucose, HCO_3_, insulin | Fluid, glucose, insulin |
| Outcome | Recovered | Recovered | Recovered | ICU admission,  Recovered |
| Precipitating factors | Fasting | Abrupt suspension of insulin therapy | Cessation of dulaglutide coupled with fasting | Fasting, surgery, dehydration, infection |
| Methodological quality | 2 | 5 | 4 | 4 |

AG, anion gap; BG, biguanides; BHB, β-hydroxybutyrate; DM, diabetes mellitus; GLP1RAs, glucagon-like peptide-1 receptor agonists; HTN, hypertension; NA, not available; SGLT2i, sodium-glucose cotransporter 2 inhibitor; SU, sulfonyl urea

**Supplemental Table S3.** Summary of each case (continued)

|  | 61^62^ | 62^62^ | 63^62^ | 64^63^ |
| --- | --- | --- | --- | --- |
| Author, year | Misaghian-Xanthos N, 2017 | Misaghian-Xanthos N, 2017 | Misaghian-Xanthos N, 2017 | Mossler J, 2018 |
| Country | USA | USA | USA | USA |
| Type of publication | Scientific paper | Scientific paper | Scientific paper | Meeting abstract |
| Age, sex, BMI (kg/m^2^) | 37, F, NA | 53, M, 38 | 66, F, 16 | 62, F, NA |
| Type of DM | T2 | T2 | T1 | T2 |
| HbA1c (%) | NA | NA | NA | NA |
| SGLT2i | Canagliflozin | Canagliflozin | Canagliflozin | Canagliflozin |
| Other anti-diabetic drugs | Insulin, BG, SU | Insulin, BG | Insulin | BG |
| Purpose of SGLT2i | DM | DM | DM | DM |
| Duration of SGLT2i treatment | NA | NA | Within 1mo | NA |
| Comorbidities | NA | NA | NA | HTN, CAD |
| Surgery | Gastric bypass for obesity | Gastric bypass for obesity | Hip surgery | Decompressive laminectomy |
| Urgency | NA | NA | NA | Elective |
| Anesthesia | NA | NA | NA | NA |
| Preop fasting | NA | NA | NA | NA |
| Preop insulin, glucose | NA | Insulin reduced | NA | NA |
| Intraop insulin, glucose | NA | NA | NA | NA |
| Postop insulin, glucose | NA | NA | NA | NA |
| Preop SGLT2i cessation | NA | NA | NA | 1d |
| Postop SGLT2i restart | NA | NA | NA | 2d postop |
| Time to presentation | On the day of surgery | 4d postop | Within 1mo | 4d postop |
| Trigger of diagnosis | NA | Arrhythmia, fatigue, malaise, Afib | NA | Nausea, confusion |
| pH,  HCO_3_ (mEq/L),  AG (mEq/L),  PCO_2_ (mmHg) | 7.12  9  19  11 | 7.23  11  16  31 | 7.16  20  21  26 | 7.14  5  32  NA |
| Urine ketones: UK, Blood ketones: BK | UK: NA  BK: positive | UK: NA  BK: positive | UK: positive  BK: NA | UK: negative  BK: BHB 3.2 mmol/L |
| Blood glucose: BG  Urine glucose: UG | BG: 162 mg/dL  UG: NA | BG: 205 mg/dL  UG: NA | BG: 337 mg/dL  UG: NA | BG: 150 mg/dL  UG: positive |
| Treatment | NA | NA | NA | Fluid, glucose, insulin |
| Outcome | NA | ICU admission | ICU admission | NA |
| Precipitating factors | Surgery | NA | NA | NA |
| Methodological quality | 3 | 3 | 2 | 4 |

AG, anion gap; BG, biguanides; BHB, β-hydroxybutyrate; CAD, coronary artery disease; DM, diabetes mellitus; HTN, hypertension; NA, not available; SGLT2i, sodium-glucose cotransporter 2 inhibitor; SU, sulfonyl urea

**Supplemental Table S3.** Summary of each case (continued)

|  | 65^64^ | 66^64^ | 67^65^ | 68^66^ |
| --- | --- | --- | --- | --- |
| Author, year | Noubleau AM, 2018 | Noubleau AM, 2018 | Osafehinti DA, 2021 | Pang J, 2018 |
| Country | USA | USA | USA | UK |
| Type of publication | Meeting abstract | Meeting abstract | Scientific paper | Meeting abstract |
| Age, sex, BMI (kg/m^2^) | 40, F, 41 | 45, F, 36 | 60, M, NA | 42, F, NA |
| Type of DM | T2 | T2 | T2 | T2 |
| HbA1c (%) | 6.5 | 8.4 | 9.6 | NA |
| SGLT2i | Canagliflozin | Canagliflozin | Empagliflozin | Canagliflozin |
| Other anti-diabetic drugs | BG, GLP1RAs | BG, GLP1RAs | BG, GLP1RAs, SU | NA |
| Purpose of SGLT2i | DM | DM | DM | DM |
| Duration of SGLT2i treatment | NA | NA | Long period | NA |
| Comorbidities | HTN | HTN | CAD | HTN, CVD |
| Surgery | Roux-en-Y gastric bypass for obesity | Laparoscopic sleeve gastrectomy for obesity | CABG | Hysterectomy |
| Urgency | Elective | Elective | Elective | Elective |
| Anesthesia | NA | NA | NA | NA |
| Preop fasting | NA | NA | NA | NA |
| Preop insulin, glucose | NA | NA | NA | NA |
| Intraop insulin, glucose | NA | NA | NA | NA |
| Postop insulin, glucose | NA | NA | NA | NA |
| Preop SGLT2i cessation | 2d | 1d | 2d (42h) | NA |
| Postop SGLT2i restart | None | None | None | 1d postop |
| Time to presentation | 1d postop | 1d postop | On the day of surgery | 2d postop |
| Trigger of diagnosis | Dyspnea, abdominal pain | Tachycardia, abdominal pain | Laboratory data | Breathe shortness, dyspnea, tachypnea, tachycardia |
| pH,  HCO_3_ (mEq/L),  AG (mEq/L),  PCO_2_ (mmHg) | 7.12  5  16  9 | 7.24  9  16  NA | 7.275  15  25  NA | 6.90  2  NA  NA |
| Urine ketones: UK, Blood ketones: BK | UK: NA  BK: BHB 9.0 mmol/L | UK: NA  BK: BHB 4.5 mmol/L | UK: NA  BK: BHB 6.5 mmol/L | UK: positive  BK: NA |
| Blood glucose: BG  Urine glucose: UG | BG: 121 mg/dL  UG: NA | BG: 116 mg/dL  UG: NA | BG: 138 mg/dL  UG: NA | BG: NA  UG: NA |
| Treatment | Fluid, glucose, insulin | Fluid, glucose, insulin | Fluid, glucose, insulin | Fluid, HCO_3_, insulin |
| Outcome | ICU admission,  Recovered | ICU admission,  Recovered | Recovered | ICU admission,  Recovered |
| Precipitating factors | Preoperative low-carbohydrate diet and perioperative NPO status | Preoperative low-carbohydrate diet and perioperative NPO status | NA | NA |
| Methodological quality | 5 | 5 | 5 | 4 |

AG, anion gap; BG, biguanides; BHB, β-hydroxybutyrate; CABG, coronary artery bypass grafting; CAD, coronary artery disease; CVD, cerebrovascular disease; DM, diabetes mellitus; GLP1RAs, glucagon-like peptide-1 receptor agonists; HTN, hypertension; NA, not available; NPO, nil per os; SGLT2i, sodium-glucose cotransporter 2 inhibitor; SU, sulfonyl urea

**Supplemental Table S3.** Summary of each case (continued)

|  | 69^67^ | 70^68^ | 71^68^ | 72^69^ |
| --- | --- | --- | --- | --- |
| Author, year | Peters AL, 2015 | Pontes JP, 2021 | Pontes JP, 2021 | Rafey MF, 2019 |
| Country | USA | Brazil | Brazil | Ireland |
| Type of publication | Scientific paper | Scientific paper | Scientific paper | Scientific paper |
| Age, sex, BMI (kg/m^2^) | 58, M, 26 | 67, M, NA | 57, M, NA | 44, M, 39 |
| Type of DM | T2 | NA | NA | T2 |
| HbA1c (%) | 9.8 | 8 | 7.7 | 8.3 |
| SGLT2i | Canagliflozin | Empagliflozin | Dapagliflozin | Canagliflozin |
| Other anti-diabetic drugs | NA | NA | Insulin | GLP1RAs |
| Purpose of SGLT2i | DM | DM | DM | DM |
| Duration of SGLT2i treatment | NA | NA | NA | NA |
| Comorbidities | NA | NA | NA | None |
| Surgery | Sigmoid colectomy | Bentall | CABG | Cervical decompression |
| Urgency | Elective | Elective | Elective | NA |
| Anesthesia | NA | NA | NA | NA |
| Preop fasting | NA | 1d | 1d | NA |
| Preop insulin, glucose | NA | NA | NA | NA |
| Intraop insulin, glucose | NA | NA | Intraop insulin | NA |
| Postop insulin, glucose | NA | NA | NA | NA |
| Preop SGLT2i cessation | NA | 1d | 1d | NA |
| Postop SGLT2i restart | NA | NA | NA | None |
| Time to presentation | 7d postop | 1d postop | 1d postop | 6d postop |
| Trigger of diagnosis | Tachypnea, vomiting, abdominal pain | Laboratory data,  Tachypnea, polyuria | Laboratory data,  Tachypnea, polyuria | Vomiting, fatigue, malaise |
| pH,  HCO_3_ (mEq/L),  AG (mEq/L),  PCO_2_ (mmHg) | 7.12  10  17  NA | 7.25  12.3  27  28.4 | 7.21  9.4  30  24 | 7.1  4.8  33.8  18.8 |
| Urine ketones: UK, Blood ketones: BK | UK: positive  BK: NA | UK: ++++  BK: not measured | UK: ++++  BK: not measured | UK: ++++  BK: 4.3 mmol/L |
| Blood glucose: BG  Urine glucose: UG | BG: 150 mg/dL  UG: NA | BG: 134-228 mg/dL  UG: ++++ | BG: 151-248 mg/dL  UG: ++++ | BG: 169.2  UG: ++ |
| Treatment | Fluid, insulin | Fluid, insulin | Fluid, HCO_3_, insulin | Fluid, insulin |
| Outcome | ICU admission | Recovered | Recovered | ICU admission,  Recovered |
| Precipitating factors | NA | CPB | CPB | NA |
| Methodological quality | 4 | 4 | 4 | 5 |

AG, anion gap; BHB, β-hydroxybutyrate; CABG, coronary artery bypass grafting; CPB, cardiopulmonary bypass; DM, diabetes mellitus; GLP1RAs, glucagon-like peptide-1 receptor agonists; HTN, hypertension; NA, not available; SGLT2i, sodium-glucose cotransporter 2 inhibitor

**Supplemental Table S3.** Summary of each case (continued)

|  | 73^69^ | 74^70^ | 75^71^ | 76^72^ |
| --- | --- | --- | --- | --- |
| Author, year | Rafey MF, 2019 | Sampani E, 2020 | Seger CD, 2021 | Seki H, 2022 |
| Country | Ireland | Greece | USA | Japan |
| Type of publication | Scientific paper | Scientific paper | Scientific paper | Scientific paper |
| Age, sex, BMI (kg/m^2^) | 59, F, 39 | 51, F, NA | 55, M, NA | 83, F, NA |
| Type of DM | T2 | T2 | T2 | Non-diabetic |
| HbA1c (%) | 9.4 | NA | 9.4 | 5.4 |
| SGLT2i | Empagliflozin | Empagliflozin | Empagliflozin | Dapagliflozin |
| Other anti-diabetic drugs | Insulin, GLP1RAs | BG, DPP4i | BG, DPP4i | None |
| Purpose of SGLT2i | DM | DM | DM | HF |
| Duration of SGLT2i treatment | 5mo | NA | NA | 67d |
| Comorbidities | None | None | NA | CAD, renal dysfunction, CVD |
| Surgery | Laparoscopic partial nephrectomy | Hysterectomy | Craniotomy and brain tumor resection | TAVR |
| Urgency | Elective | Elective | Elective | Elective |
| Anesthesia | NA | NA | GA | GA |
| Preop fasting | NA | NA | 1d | 1d |
| Preop insulin, glucose | NA | NA | NA | NA |
| Intraop insulin, glucose | NA | NA | Intraoperative glucose and insulin for EDKA treatment | Intraoperative glucose |
| Postop insulin, glucose | NA | NA | None | NA |
| Preop SGLT2i cessation | None | NA | None | None |
| Postop SGLT2i restart | None | NA | None | Discontinued on 1d postop |
| Time to presentation | 3d postop | 6d postop | Intraop | 1d postop |
| Trigger of diagnosis | Dyspnea, dizziness | Tachypnea, vomiting, abdominal pain, fatigue, malaise | Laboratory data | Laboratory data |
| pH,  HCO_3_ (mEq/L),  AG (mEq/L),  PCO_2_ (mmHg) | 7.23  9.3  32.6  NA | 7.05  3  16.9  12 | 7.24  16.7  22  NA | 7.265  15.9  13.8  36.3 |
| Urine ketones: UK, Blood ketones: BK | UK: +++  BK: 4.8 mmol/L | UK: 27.5 mmol/L  BK: 1000 mmol/L | UK: NA  BK: BHB 8.5 mmol/L | UK: ++++  BK: NA |
| Blood glucose: BG  Urine glucose: UG | BG: 221.4 mg/dL  UG: ++ | BG: 121 mg/dL  UG: NA | BG: 142 mg/dL  UG: NA | BG: 52 mg/dL  UG: ++ |
| Treatment | Fluid, insulin | Fluid, HCO_3_, insulin | Fluid, glucose, insulin | Fluid, glucose |
| Outcome | Recovered | Recovered | Recovered | Recovered |
| Precipitating factors | NA | Postoperative fasting, infection, surgical stress, dehydration | Perioperative fasting | Perioperative fasting |
| Methodological quality | 5 | 4 | 5 | 5 |

AG, anion gap; BG, biguanides; BHB, β-hydroxybutyrate; CAD, coronary artery disease; CVD, cerebrovascular disease; DM, diabetes mellitus; DPP4i, dipeptidyl peptidase-4 inhibitor; GLP1RAs, glucagon-like peptide-1 receptor agonists; HTN, hypertension; NA, not available; SGLT2i, sodium-glucose cotransporter 2 inhibitor; TAVR, transcatheter aortic valve replacement

**Supplemental Table S3.** Summary of each case (continued)

|  | 77^73^ | 78^73^ | 79^74^ | 80^75^ |
| --- | --- | --- | --- | --- |
| Author, year | Shah M, 2022 | Shah M, 2022 | Tansey DJ, 2019 | Theodore D, 2018 |
| Country | India | India | Ireland | USA |
| Type of publication | Scientific paper | Scientific paper | Meeting abstract | Scientific paper |
| Age, sex, BMI (kg/m^2^) | 74, F, NA | 71, F, NA | 53, F, NA | 79, M, NA |
| Type of DM | T2 | T2 | T2 | T2 |
| HbA1c (%) | NA | NA | NA | 7.1 |
| SGLT2i | Empagliflozin | Empagliflozin | Empagliflozin | Canagliflozin |
| Other anti-diabetic drugs | DPP4i | NA | BG, DPP4i | NA |
| Purpose of SGLT2i | DM | DM | DM | DM |
| Duration of SGLT2i treatment | 5y | NA | NA | NA |
| Comorbidities | NA | NA | NA | HTN, renal dysfunction |
| Surgery | CABG | Surgery for large bowel obstruction | Cholecystectomy | Extension of spinal fusion and rod replacement |
| Urgency | Elective | Emergency | Elective | NA |
| Anesthesia | NA | NA | NA | NA |
| Preop fasting | NA | NA | NA | NA |
| Preop insulin, glucose | NA | NA | NA | NA |
| Intraop insulin, glucose | NA | NA | NA | NA |
| Postop insulin, glucose | NA | NA | NA | NA |
| Preop SGLT2i cessation | 1d | NA | NA | NA |
| Postop SGLT2i restart | None | NA | NA | NA |
| Time to presentation | On the day of surgery | 1d postop | 3d postop | On the day of surgery |
| Trigger of diagnosis | Laboratory data | Laboratory data | Nausea | Laboratory data |
| pH,  HCO_3_ (mEq/L),  AG (mEq/L),  PCO_2_ (mmHg) | High anion gap metabolic acidosis  NA  22  NA | High anion gap metabolic acidosis  NA  16  NA | 7.04  4.3  18  16.2 | 7.26  16.9  13  38.2 |
| Urine ketones: UK, Blood ketones: BK | UK: 27.5 mmol/L  BK: NA | UK: 27.5 mmol/L  BK: 2.8 mmol/l | UK: NA  BK: 4.5 mmol/L | UK: positive  BK: moderate |
| Blood glucose: BG  Urine glucose: UG | BG: 216 mg/dL  UG: ++ | BG: 186 mg/dL  UG: ++ | BG: 234 mg/dL  UG: NA | BG: 150 mg/dL  UG: NA |
| Treatment | Fluid, glucose, insulin | DKA protocol | DKA protocol | Fluid, glucose, insulin |
| Outcome | Recovered | Recovered | ICU admission  Recovered | ICU admission, mechanical ventilation  Recovered |
| Precipitating factors | Surgical stress | Surgical stress | NA | NPO the night before |
| Methodological quality | 2 | 3 | 5 | 5 |

AG, anion gap; BG, biguanides; BHB, β-hydroxybutyrate; CABG, coronary artery bypass grafting; DM, diabetes mellitus; DPP4i, dipeptidyl peptidase-4 inhibitor; HTN, hypertension; NA, not available; SGLT2i, sodium-glucose cotransporter 2 inhibitor

**Supplemental Table S3.** Summary of each case (continued)

|  | 81^76^ | 82^76^ | 83^77^ | 84^78^ |
| --- | --- | --- | --- | --- |
| Author, year | Tsai MK, 2019 | Tsai MK, 2019 | Ullah S, 2016 | Vadi S |
| Country | Taiwan | Taiwan | USA | India |
| Type of publication | Scientific paper | Scientific paper | Scientific paper | Scientific paper |
| Age, sex, BMI (kg/m^2^) | 60, F, NA | 63, F, NA | 40, F, NA | 56, M, 41 |
| Type of DM | T2 | T2 | T2 | T2 |
| HbA1c (%) | 12 | 11 | NA | NA |
| SGLT2i | Dapagliflozin | Dapagliflozin | Canagliflozin | Dapagliflozin |
| Other anti-diabetic drugs | BG, DPP4i | BG, SU | NA | Insulin, AGIs, BG |
| Purpose of SGLT2i | DM | DM | DM | DM |
| Duration of SGLT2i treatment | 1mo | Several years | 1y | 5y |
| Comorbidities | None | NA | None | HTN |
| Surgery | ORIF for tibia fraction, spinal fusion | ORIF for humerus and femur fracture | Incision and drainage for cellulitis | CABG |
| Urgency | NA | NA | Emergency | Elective |
| Anesthesia | NA | NA | NA | NA |
| Preop fasting | NA | NA | NA | NA |
| Preop insulin, glucose | NA | NA | NA | Preop insulin |
| Intraop insulin, glucose | NA | NA | NA | NA |
| Postop insulin, glucose | NA | NA | NA | NA |
| Preop SGLT2i cessation | NA | NA | None | 2d |
| Postop SGLT2i restart | NA | NA | NA | NA |
| Time to presentation | 11d postop | 5d postop | 2d postop | 1d postop |
| Trigger of diagnosis | Dyspnea | Dyspnea, vomiting, dizziness | Laboratory data | Excessive thirst |
| pH,  HCO_3_ (mEq/L),  AG (mEq/L),  PCO_2_ (mmHg) | 7.06  2  24  NA | 7.15  6.3  23  NA | 7.2  8  18  21 | 7.26  15.6  19.3  NA |
| Urine ketones: UK, Blood ketones: BK | UK: NA  BK: 5.6 mmol/L | UK: NA  BK: 3.8 mmol/L | UK: NA  BK: 8.9 mmol/L | UK: NA  BK: 0.81 mmol/L |
| Blood glucose: BG  Urine glucose: UG | BG: 116-182 mg/dL  UG: NA | BG: 194-338 mg/dL  UG: NA | BG: >250 mg/dL  UG: NA | BG: 145-249 mg/dL  UG: NA |
| Treatment | Fluid, insulin | Fluid, insulin | Fluid, HCO_3_ | Insulin |
| Outcome | ICU admission  Recovered | ICU admission, mechanical ventilation  Recovered | ICU admission  Recovered | ICU admission  Recovered |
| Precipitating factors | NA | NA | NA | NA |
| Methodological quality | 5 | 5 | 5 | 5 |

AG, anion gap; AGIs , alpha glucosidase inhibitors; BG, biguanides; BHB, β-hydroxybutyrate; CABG, coronary artery bypass grafting; DM, diabetes mellitus; DPP4i, dipeptidyl peptidase-4 inhibitor; HTN, hypertension; NA, not available; ORIF, open reduction and internal fixation; SGLT2i, sodium-glucose cotransporter 2 inhibitor; SU, sulfonyl urea

**Supplemental Table S3.** Summary of each case (continued)

|  | 85^79^ | 86^80^ | 87^81^ | 88^82^ |
| --- | --- | --- | --- | --- |
| Author, year | Van Niekerk C, 2018 | Wang KM, 2020 | Wang Q, 2022 | Wang R, 2021 |
| Country | USA | USA | China | Australia |
| Type of publication | Scientific paper | Scientific paper | Scientific paper | Scientific paper |
| Age, sex, BMI (kg/m^2^) | 52, M, 45 | 40s, F, NA | 57, F, NA | NA, NA, NA |
| Type of DM | T2 | T2 | T2 | NA |
| HbA1c (%) | 7.4 | NA | NA | NA |
| SGLT2i | Canagliflozin | Empagliflozin | Dapagliflozin | Empagliflozin or dapagliflozin |
| Other anti-diabetic drugs | BG | BG, TZD | BG | NA |
| Purpose of SGLT2i | DM | DM | DM | DM |
| Duration of SGLT2i treatment | NA | NA | NA | NA |
| Comorbidities | HTN | CVD | HTN | NA |
| Surgery | Laparoscopic Roux-en-Y gastric bypass for obesity | Cerebral revascularization | Laparoscopic distal pancreatectomy, emergency laparotomy POD9 from first operation | CABG |
| Urgency | Elective | Elective | Elective and emergency | Emergency |
| Anesthesia | NA | NA | NA | NA |
| Preop fasting | 1d | NA | NA | 5h |
| Preop insulin, glucose | NA | NA | NA | NA |
| Intraop insulin, glucose | NA | NA | NA | NA |
| Postop insulin, glucose | NA | NA | NA | NA |
| Preop SGLT2i cessation | 1d | 18h | NA | 1d (24h) |
| Postop SGLT2i restart | NA | NA | NA | NA |
| Time to presentation | 2d postop | 1d postop | 9d postop | NA |
| Trigger of diagnosis | Laboratory data | Laboratory data | Tachypnea, tachycardia, vomiting, abdominal pain | NA |
| pH,  HCO_3_ (mEq/L),  AG (mEq/L),  PCO_2_ (mmHg) | 7.19  9  23  24 | 7.01  NA  17  11.5 | 7.09  17  36.2  56.7 | 7.28  22  NA  50.3 |
| Urine ketones: UK, Blood ketones: BK | UK: NA  BK: BHB 4.8 mmol/L | UK: ++++  BK: BHB 7.7 mmol/L | UK: strong positive  BK: BHB 10.9 mmol/L | UK: NA  BK: BHB 4.8 mmol/l |
| Blood glucose: BG  Urine glucose: UG | BG: 112 mg/dL  UG: NA | BG: 149 mg/dL  UG: ++ | BG: 270 mg/dL  UG: NA | BG: 162 mg/dL  UG: NA |
| Treatment | Fluid, glucose, insulin | Fluid, glucose, insulin | Fluid, insulin | Fluid, glucose, insulin |
| Outcome | Recovered | ICU admission  Death | ICU admission  Recovered | NA |
| Precipitating factors | NA | NA | NA | NA |
| Methodological quality | 5 | 5 | 5 | 3 |

AG, anion gap; BG, biguanides; BHB, β-hydroxybutyrate; CABG, coronary artery bypass grafting; CVD, cerebrovascular disease; DM, diabetes mellitus; HTN, hypertension; NA, not available; SGLT2i, sodium-glucose cotransporter 2 inhibitor; TZD, thiazolidines

**Supplemental Table S3.** Summary of each case (continued)

|  | 89^82^ | 90^82^ | 91^82^ | 92^82^ |
| --- | --- | --- | --- | --- |
| Author, year | Wang R, 2021 | Wang R, 2021 | Wang R, 2021 | Wang R, 2021 |
| Country | Australia | Australia | Australia | Australia |
| Type of publication | Scientific paper | Scientific paper | Scientific paper | Scientific paper |
| Age, sex, BMI (kg/m^2^) | NA, NA, NA | NA, NA, NA | NA, NA, NA | NA, NA, NA |
| Type of DM | NA | NA | NA | NA |
| HbA1c (%) | NA | NA | NA | NA |
| SGLT2i | Empagliflozin or dapagliflozin | Empagliflozin or dapagliflozin | Empagliflozin or dapagliflozin | Empagliflozin or dapagliflozin |
| Other anti-diabetic drugs | NA | NA | NA | NA |
| Purpose of SGLT2i | DM | DM | DM | DM |
| Duration of SGLT2i treatment | NA | NA | NA | NA |
| Comorbidities | NA | NA | NA | NA |
| Surgery | Laminectomy | Craniotomy | Bowel resection | Endovascular clot retrieval |
| Urgency | Emergency | Emergency | Emergency | Emergency |
| Anesthesia | NA | NA | NA | NA |
| Preop fasting | 12h | 12h | 16h | 16h |
| Preop insulin, glucose | NA | NA | NA | NA |
| Intraop insulin, glucose | Intraop insulin | NA | Intraop insulin | NA |
| Postop insulin, glucose | NA | Postop insulin | NA | Postop insulin |
| Preop SGLT2i cessation | 1d (24h) | 1d (24h) | 2d (30h) | 2d (31h) |
| Postop SGLT2i restart | NA | NA | NA | NA |
| Time to presentation | Intraop | Postop | Intraop | Postop |
| Trigger of diagnosis | NA | NA | NA | NA |
| pH,  HCO_3_ (mEq/L),  AG (mEq/L),  PCO_2_ (mmHg) | 7.29  20  NA  42.8 | 7.26  17  NA  33 | 7.28  28  NA  39.8 | 7.23  23  NA  65.3 |
| Urine ketones: UK, Blood ketones: BK | UK: NA  BK: BHB 1.1 mmol/L | UK: NA  BK: BHB 6.5 mmol/L | UK: NA  BK: BHB 4.8 mmol/L | UK: NA  BK: BHB 1.8 mmol/L |
| Blood glucose: BG  Urine glucose: UG | BG: 135 mg/dL  UG: NA | BG: 207 mg/dL  UG: NA | BG: 201.6 mg/dL  UG: NA | BG: 255.6 mg/dL  UG: NA |
| Treatment | Fluid, glucose, insulin | Fluid, glucose, insulin | Fluid, glucose, insulin | Fluid, glucose, insulin |
| Outcome | NA | NA | NA | NA |
| Precipitating factors | NA | NA | NA | NA |
| Methodological quality | 3 | 3 | 3 | 3 |

AG, anion gap; β-hydroxybutyrate; DM, diabetes mellitus; NA, not available; SGLT2i, sodium-glucose cotransporter 2 inhibitor

**Supplemental Table S3.** Summary of each case (continued)

|  | 93^82^ | 94^82^ | 95^83^ | 96^84^ |
| --- | --- | --- | --- | --- |
| Author, year | Wang R, 2021 | Wang R, 2021 | Wohlrab P, 2021 | Wong YC, 2021 |
| Country | Australia | Australia | Austria | Taiwan |
| Type of publication | Scientific paper | Scientific paper | Scientific paper | Scientific paper |
| Age, sex, BMI (kg/m^2^) | NA, NA, NA | NA, NA, NA | 62, M, NA | 57, F, NA |
| Type of DM | NA | NA | T2 | T2 |
| HbA1c (%) | NA | NA | NA | NA |
| SGLT2i | Empagliflozin or dapagliflozin | Empagliflozin or dapagliflozin | Empagliflozin | Dapagliflozin |
| Other anti-diabetic drugs | NA | NA | NA | BG |
| Purpose of SGLT2i | DM | DM | DM | DM |
| Duration of SGLT2i treatment | NA | NA | NA | 1y |
| Comorbidities | NA | NA | HTN | NA |
| Surgery | CABG | Laparoscopic hysterectomy | CABG | ORIF for femoral proximal shaft fracture |
| Urgency | Elective | Elective | Elective | Emergency |
| Anesthesia | NA | NA | NA | GA |
| Preop fasting | 14h | 12h | NA | NA |
| Preop insulin, glucose | NA | NA | Preop insulin | Preop glucose |
| Intraop insulin, glucose | NA | NA | Intraop insulin | NA |
| Postop insulin, glucose | Postop insulin | Postop insulin | NA | NA |
| Preop SGLT2i cessation | None (1h) | None (5h) | 1d | 1d |
| Postop SGLT2i restart | NA | NA | NA | None |
| Time to presentation | Postop | Postop | 1d postop | 2d postop |
| Trigger of diagnosis | NA | NA | Laboratory data, tachypnea, nausea, abdominal pain | Tachypnea, dyspnea, thirst |
| pH,  HCO_3_ (mEq/L),  AG (mEq/L),  PCO_2_ (mmHg) | 7.2  14  NA  33 | 7.27  22  NA  48.8 | 7.1  13.3  12  35 | 6.974  4.2  NA  17.8 |
| Urine ketones: UK, Blood ketones: BK | UK: NA  BK:BHB 6.4 mmol/L | UK: NA  BK: BHB 0.9 mmol/L | UK: positive  BK: NA | UK: ++++  BK: BHB 4.5 mmol/L |
| Blood glucose: BG  Urine glucose: UG | BG: 216 mg/dL  UG: NA | BG: 131.4 mg/dL  UG: NA | BG: 148 mg/dL  UG: NA | BG: 187 mg/dL  UG: NA |
| Treatment | Fluid, glucose, insulin | Fluid, insulin | Fluid, glucose, insulin | Fluid, glucose, insulin |
| Outcome | NA | NA | ICU admission  Recovered | ICU admission, mechanical ventilation, dialysis  Recovered |
| Precipitating factors | NA | NA | NA | NA |
| Methodological quality | 3 | 3 | 5 | 5 |

AG, anion gap; BHB, β-hydroxybutyrate; CABG, coronary artery bypass grafting; DM, diabetes mellitus; GA, general anesthesia; HTN, hypertension; NA, not available; ORIF, open reduction and internal fixation; SGLT2i, sodium-glucose cotransporter 2 inhibitor

**Supplemental Table S3.** Summary of each case (continued)

|  | 97^85^ | 98^86^ | 99^87^ |
| --- | --- | --- | --- |
| Author, year | Wood T, 2016 | Yared KE, 2021 | Zhang L, 2018 |
| Country | Canada | USA | Canada |
| Type of publication | Scientific paper | Scientific paper | Scientific paper |
| Age, sex, BMI (kg/m^2^) | 74, M, NA | 49, M, 48 | 70, M, NA |
| Type of DM | T2 | T2 | T2 |
| HbA1c (%) | NA | 7.6 | 7.8 |
| SGLT2i | Canagliflozin | Empagliflozin | Empagliflozin |
| Other anti-diabetic drugs | None | Insulin, BG, GLP1RAs | BG, GLP1RAs, SU |
| Purpose of SGLT2i | DM | DM | DM |
| Duration of SGLT2i treatment | 8mo | NA | A few years |
| Comorbidities | HTN | NA | CAD |
| Surgery | Pancreaticoduodenectomy | Laparoscopic Roux-en-Y gastric bypass for obesity | CABG |
| Urgency | Elective | Elective | NA |
| Anesthesia | NA | NA | NA |
| Preop fasting | NA | NA | NA |
| Preop insulin, glucose | NA | NA | NA |
| Intraop insulin, glucose | NA | NA | NA |
| Postop insulin, glucose | Insulin sliding scale | NA | Postop insulin |
| Preop SGLT2i cessation | 1d | None | NA |
| Postop SGLT2i restart | None | Not discontinued | None |
| Time to presentation | 10d postop | 3d postop | NA |
| Trigger of diagnosis | Laboratory data | Breath shortness, dyspnea, headache | Nausea, vomiting, fatigue, malaise |
| pH,  HCO_3_ (mEq/L),  AG (mEq/L),  PCO_2_ (mmHg) | 7.24  9  23  23 | 7.106  17.6  29  NA | 7.27  10  31  NA |
| Urine ketones: UK, Blood ketones: BK | UK: 15.6 mmol/L  BK: 9 mmol/L | UK: NA  BK: BHB>7.7 mmol/L | UK: positive  BK: BHB 3.2 mmol/L |
| Blood glucose: BG  Urine glucose: UG | BG: 122.4-189 mg/dL  UG: NA | BG: 180 mg/dL  UG: ++++ | BG: 201.6 mg/dL  UG: NA |
| Treatment | Fluid, HCO_3_, insulin | Fluid, glucose, insulin | Fluid, insulin |
| Outcome | Recovered | Recovered | Recovered |
| Precipitating factors | Postoperative AKI | A very low-calorie liquid diet | Pneumonia |
| Methodological quality | 5 | 5 | 5 |

AG, anion gap; AKI, acute kidney injury; BG, biguanide; BHB, β-hydroxybutyrate; CAD, coronary artery disease; CABG, coronary artery bypass grafting; DM, diabetes mellitus; GLP1RAs, glucagon-like peptide-1 receptor agonists; HTN, hypertension; NA, not available; SGLT2i, sodium-glucose cotransporter 2 inhibitor; SU, sulfonyl urea

**Supplemental Table S4.** Details of preoperative diabetes medication

| Diabetic regimen | Reported cases |
| --- | --- |
| SGLT2i alone | 1 |
|  |  |
| SGLT2i + BG | 18 |
| SGLT2i + GLP1RAs | 2 |
| SGLT2i + DPP4 i | 1 |
|  |  |
| SGLT2i + BG + DPP4i | 9 |
| SGLT2i + BG + GLP1RAs | 9 |
| SGLT2i + BG + SU | 3 |
| SGLT2i + BG + TZDs | 1 |
|  |  |
| SGLT2i + BG + SU + DPP4i | 2 |
| SGLT2i + BG + SU + GLP1RAs | 2 |
| SGLT2i + BG + DPP4i + TZDs | 1 |
|  |  |
| SGLT2i + insulin | 6 |
|  |  |
| SGLT2i + insulin + BG | 11 |
| SGLT2i + insulin + GLP1RAs | 2 |
|  |  |
| SGLT2i + insulin + BG + SU | 3 |
| SGLT2i + insulin + BG + DPP4i | 1 |
| SGLT2i + insulin + BG + GLP1RAs | 1 |
|  |  |
| SGLT2i + insulin+ BG + GLP1Ras + AGIs | 1 |
|  |  |
| Not reported | 24 |
| Unclear | 1 |

AGIs, alpha glucosidase inhibitors; BG, biguanide; DPP4i, Dipeptidyl Peptidase-4 Inhibitor; GLP1RAs, glucagon-like peptide-1 receptor agonists; SGLT2i, sodium-glucose cotransporter 2 inhibitor; SU, sulfonyl urea; TZDs, thiazolidines

**Supplemental Table S5.** Type of surgery in reported cases

| Surgical site | | Type of surgery | Number of reported cases |
| --- | --- | --- | --- |
| Digestive | Upper gastrointestinal | Roux-en-Y gastric bypass (bariatric surgery) | 13 |
|  |  | Sleeve gastrectomy (bariatric surgery) | 11 |
|  |  | Cholecystectomy | 5 |
|  |  | Appendectomy | 3 |
|  |  | Gastrojejunal bypass | 1 |
|  | Colorectal | Small-bowel resection | 1 |
|  |  | Sigmoid colectomy | 1 |
|  |  | Bowel resection | 1 |
|  | Others | Distal pancreatectomy | 1 |
|  |  | Partial pancreatic resection | 1 |
|  |  | Pancreaticoduodenectomy | 1 |
|  |  | Emergency laparotomy after laparoscopic distal pancreatectomy | 1 |
|  |  | Gastric wedge resection for gastric perforation | 1 |
|  |  | Unknown (large bowel obstruction) | 1 |
| Cardiovascular | Cardiac | CABG | 18 |
|  |  | Bentall | 1 |
|  |  | TAVR | 1 |
|  | Vascular | Femoral endarterectomy | 1 |
|  |  | Endovascular clot retrieval | 1 |
| Orthopedic | Spine | Laminectomy | 2 |
|  |  | Lumber spine fusion | 1 |
|  |  | Spinal fusion and laminectomy | 1 |
|  |  | Cervical decompression | 1 |
|  |  | Extension of spinal fusion and rod replacement | 1 |
|  | Hip and knee | Total knee replacement | 3 |
|  |  | Knee surgery | 1 |
|  |  | Hip surgery | 1 |
|  | Others | ORIF for intertrochanteric fracture | 1 |
|  |  | ORIF for humerus and femur fracture | 1 |
|  |  | ORIF for femoral shaft fracture | 1 |
| Neuro | Intracranial | Brain tumor resection | 2 |
|  |  | Cerebral aneurysm clopping | 1 |
|  |  | Cerebral revascularization | 1 |
|  |  | Craniotomy (details not reported) | 1 |
|  | Others | VP shunt exchange | 1 |
| Urological and gynecological |  | Hysterectomy | 3 |
|  |  | Gynecological laparotomy | 1 |
|  |  | Partial nephrectomy | 1 |
|  |  | Laparotomy after ATH for hematoma drainage | 1 |
| Soft tissue surgery |  | Abdominoplasty | 1 |
|  |  | Reconstructive surgery after breast cancer | 1 |
|  |  | Cosmetic surgery | 1 |
|  |  | Debridement | 5 |
| Others |  | Dental extraction | 1 |
| Total |  |  | 99 |

ATH, abdominal total hysterectomy; CABG, coronary artery bypass grafting; ORIF, open reduction and internal fixation; TAVR, trans-aortic valvular replacement; VP, ventriculoperitoneal

**Supplemental Table S6.** Details of perioperative fluid management

| Intervention | Timing | Administration | Number of reported cases |
| --- | --- | --- | --- |
| Glucose* | Preoperative | - | 1 |
|  |  | + | 1 |
|  |  | Not described | 97 |
|  | Intraoperative | - | 0 |
|  |  | + | 4 |
|  |  | Not described | 95 |
|  | Postoperative | - | 1 |
|  |  | + | 1 |
|  |  | Not described | 97 |
| Insulin* | Preoperative | - | 1 |
|  |  | + | 5 |
|  |  | Reduced | 3 |
|  |  | Discontinued | 1 |
|  |  | Not described | 89 |
|  | Intraoperative | - | 0 |
|  |  | + | 9 |
|  |  | Not described | 90 |
|  | Postoperative | - | 2 |
|  |  | + | 11 |
|  |  | Discontinued | 5 |
|  |  | Sliding scale | 1 |
|  |  | Not described | 80 |

*Does not include the administration for the treatment of ketoacidosis

**Supplemental Table S7.** Preoperative withholding period of the SGLT2is

| Withholding period | Number of reported cases |
| --- | --- |
| 1 day | 33 |
| 2 days | 9 |
| 18 hours | 1 |
| 42 hours | 1 |
| No cessation (took SGLT2i on the day of surgery) | 14 |
| Omitted perioperatively | 1 |
| No description | 40 |
| Total | 99 |

**Supplemental Figure S1.** Distribution of age in reported cases

**Supplemental Figure S2.** Distribution of baseline HbA1c value in reported cases


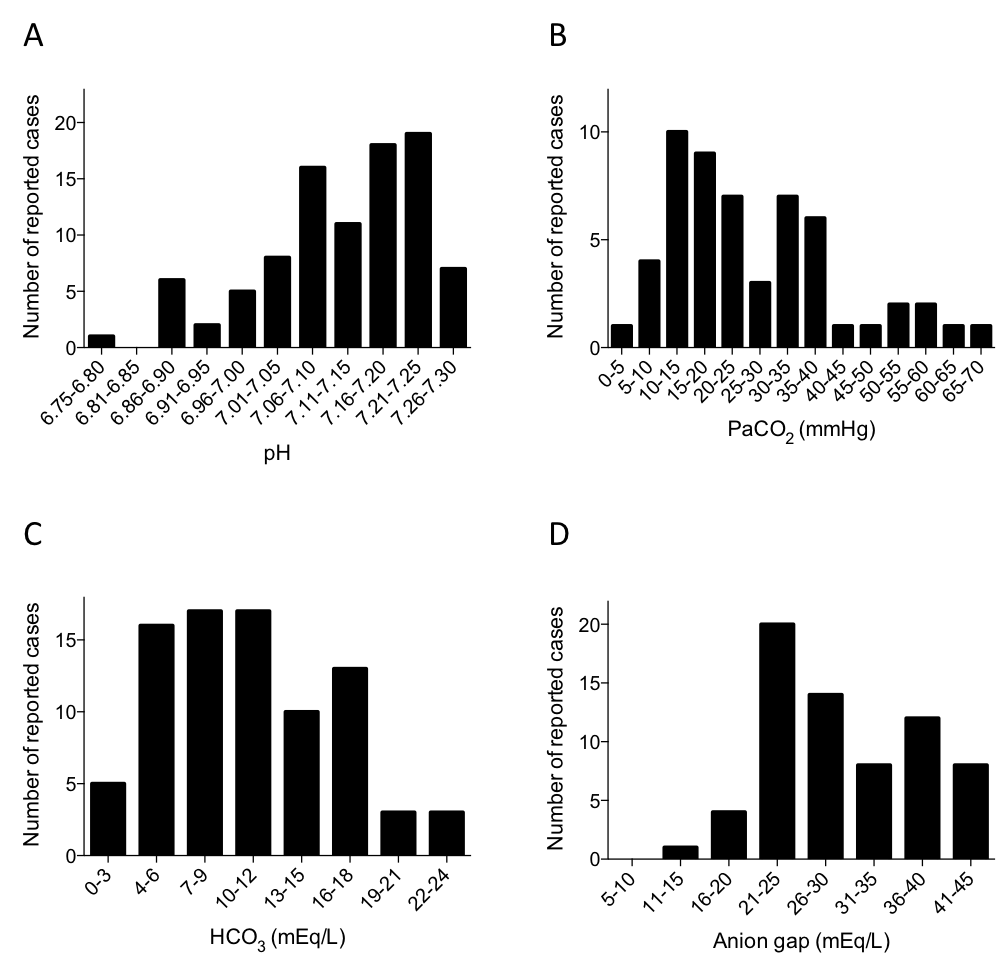


**Supplemental Figure S3.** Details of ketoacidosis. A, pH; B. PaCO_2_; C. HCO_3_; D, anion gap at the time of diagnosis

**Supplemental Figure S4**. Distribution of blood glucose level at the time of diagnosis
